# Striatal Cholinergic Interneurons Control Physical Nicotine Withdrawal via Muscarinic Receptor Signaling

**DOI:** 10.1002/advs.202402274

**Published:** 2024-11-03

**Authors:** Baeksun Kim, Han Ah Kim, Junsung Woo, Hyeon‐Jeong Lee, Tae Kyoo Kim, Hophil Min, C. Justin Lee, Heh‐In Im

**Affiliations:** ^1^ Center for Brain Function Brain Science Institute Korea Institute of Science and Technology (KIST) Seoul 02792 Republic of Korea; ^2^ Division of Bio‐Medical Science & Technology KIST School Korea National University of Science and Technology (UST) Seoul 02792 Republic of Korea; ^3^ Center for Glia‐Neuron Interaction Brain Science Institute KIST Seoul 02792 Republic of Korea; ^4^ Doping Control Center KIST Seoul 02792 Republic of Korea

**Keywords:** cholinergic interneurons, dopamine, dorsal striatum, muscarinic receptor, neural activity, nicotine withdrawal, somatic signs

## Abstract

Striatal cholinergic interneurons (ChIs) provide acetylcholine tone to the striatum and govern motor functions. Nicotine withdrawal elicits physical symptoms that dysregulate motor behavior. Here, the role of striatal ChIs in physical nicotine withdrawal is investigated. Mice under RNAi‐dependent genetic inhibition of striatal ChIs (ChI^GI^) by suppressing the sodium channel subunit Na_V_1.1, lessening action potential generation and activity‐dependent acetylcholine release is first generated. ChI^GI^ markedly reduced the somatic signs of nicotine withdrawal without affecting other nicotine‐dependent or striatum‐associated behaviors. Multielectrode array (MEA) recording revealed that ChI^GI^ reversed ex vivo nicotine‐induced alterations in the number of neural population spikes in the dorsal striatum. Notably, the drug repurposing strategy revealed that a clinically‐approved antimuscarinic drug, procyclidine, fully mimicked the therapeutic electrophysiological effects of ChI^GI^. Furthermore, both ChI^GI^ and procyclidine prevented the nicotine withdrawal‐induced reduction in striatal dopamine release in vivo. Lastly, therapeutic intervention with procyclidine dose‐dependently diminished the physical signs of nicotine withdrawal. The data demonstrated that the striatal ChIs are a critical substrate of physical nicotine withdrawal and that muscarinic antagonism holds therapeutic potential against nicotine withdrawal.

## Graphical abstract

The role of striatal cholinergic interneurons in nicotine withdrawal is explored. Inhibition of striatal cholinergic interneurons (ChIs) ameliorated nicotine‐dependent alterations in striatal neural population activity, nicotine withdrawal‐induced deficit in striatal dopamine release, and physical nicotine withdrawal. The clinically‐approved antimuscarinic drug, procyclidine, fully recapitulated the therapeutic effects of striatal ChI inhibition on physical nicotine withdrawal.

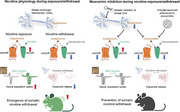

## 1 Introduction

Cholinergic interneurons (ChIs) comprise approximately 1%–2% of the striatal neuronal population yet provide dense innervation and acetylcholine tone to the local circuitry of the entire striatum.^[^ ^1^ ^]^ The striatal ChIs exert their effect by cholinergic neurotransmission‐dependent activation of the nicotinic and muscarinic acetylcholine receptors (nAChRs and mAChRs, respectively). One of the most notable and potent nAChR agonists is nicotine, the tobacco‐derived bioactive compound that holds rewarding properties and triggers dependence in animals.^[^ ^2^ ^]^ This evident connection between the striatal ChIs and nicotine implies that striatal ChIs have a role in the pathophysiology of nicotine dependence. However, the relationship between striatal ChIs and nicotine dependence has not been investigated to date.

Upon abrupt cessation of cigarette smoking, withdrawal symptoms ensue in the dependent subjects. The negative reinforcement arising from withdrawal symptoms is thought to promote continued cigarette smoking,^[^ ^3^ ^]^ and the worsening of withdrawal symptoms increases the likelihood of smoking relapse.^[^ ^4^ ^]^ Therefore, nicotine withdrawal presents a critical obstacle to smoking cessation,^[^ ^5^ ^]^ and understanding the biological mechanism of nicotine withdrawal is pivotal for the development of effective therapeutics against nicotine dependence.

Nicotine withdrawal is generally characterized by physical and affective aspects in humans.^[^ ^6^ ^]^ The rodent models effectively mimic the nicotine withdrawal‐associated behavioral symptoms in humans.^[^ ^7^ ^]^ In rodents, the physical component of nicotine withdrawal is generally marked by somatic signs and locomotor depression, while anxiety‐like behaviors and brain reward threshold elevation mark the affective component. Interestingly, both the physical and affective symptoms diminish the quality of life, but the importance of the physical symptoms of nicotine withdrawal has been relatively undervalued.

The ChIs in the dorsal striatum have been primarily implicated in movement disorders.^[^ ^8^ ^]^ Recent studies have shown that the ChIs control motor dysfunction and its associated striatal neuronal activity in the mouse models of Parkinson's disease.^[^ ^9^ ^]^ Notably, the findings unequivocally demonstrated that depletion of ChI activity or signaling in the dorsal striatum leads to the behavioral rescue of motor dysfunctions, which indicates that the striatal ChIs can be a therapeutic target for movement disorders. In light of this evidence, we hypothesized that the ChIs in the dorsal striatum have an essential role in the physical symptoms of nicotine withdrawal.

Here, we investigated the therapeutic role of striatal ChIs in the physical aspect of nicotine withdrawal through genetic, molecular, electrophysiological, neurotransmitter, and behavioral approaches. To this end, we implemented a method for genetic inhibition of neurons and a mouse model of nicotine dependence. We enforced the genetic inhibition of striatal ChIs (ChI^GI^) by miRNA‐mediated knockdown of voltage‐gated sodium channels,^[^ ^11^ ^]^ effectively lessening the neuronal activity of striatal ChIs. With the ChI^GI^ mice, we modeled nicotine withdrawal through mecamylamine‐induced precipitation of physical withdrawal signs from a low‐dose, repeated, bolus nicotine exposure regimen^[^ ^7d^ ^]^ that closely mimics the light nicotine intake during the experimentation stage of cigarette smoking.^[^ ^12^ ^]^

Briefly, we showed that the activity of striatal ChIs is essential for generating the somatic signs of nicotine withdrawal. Delving into the mechanism, we revealed that ChI^GI^ prevents the ex vivo nicotine‐evoked alterations in striatal neural activity and the in vivo nicotine withdrawal‐induced reduction in striatal dopamine release. Importantly, we showed through drug repurposing^[^ ^13^ ^]^ that a clinically‐approved centrally‐acting antimuscarinic drug, procyclidine, fully mimics the therapeutic effects of ChI^GI^ by significantly attenuating the electrophysiological, neurotransmitter, and behavioral signatures of nicotine withdrawal. Through this study, we unveil the importance of striatal cholinergic neurotransmission in the pathophysiology of nicotine dependence and propose that the striatal ChIs are a critical component of nicotine withdrawal regarding motor behavior, striatal neural activity, and dopamine dynamics.

## 2 Results

### 2.1 Genetic Inhibition of Cholinergic Interneurons by RNA Interference

**Figure 1:**
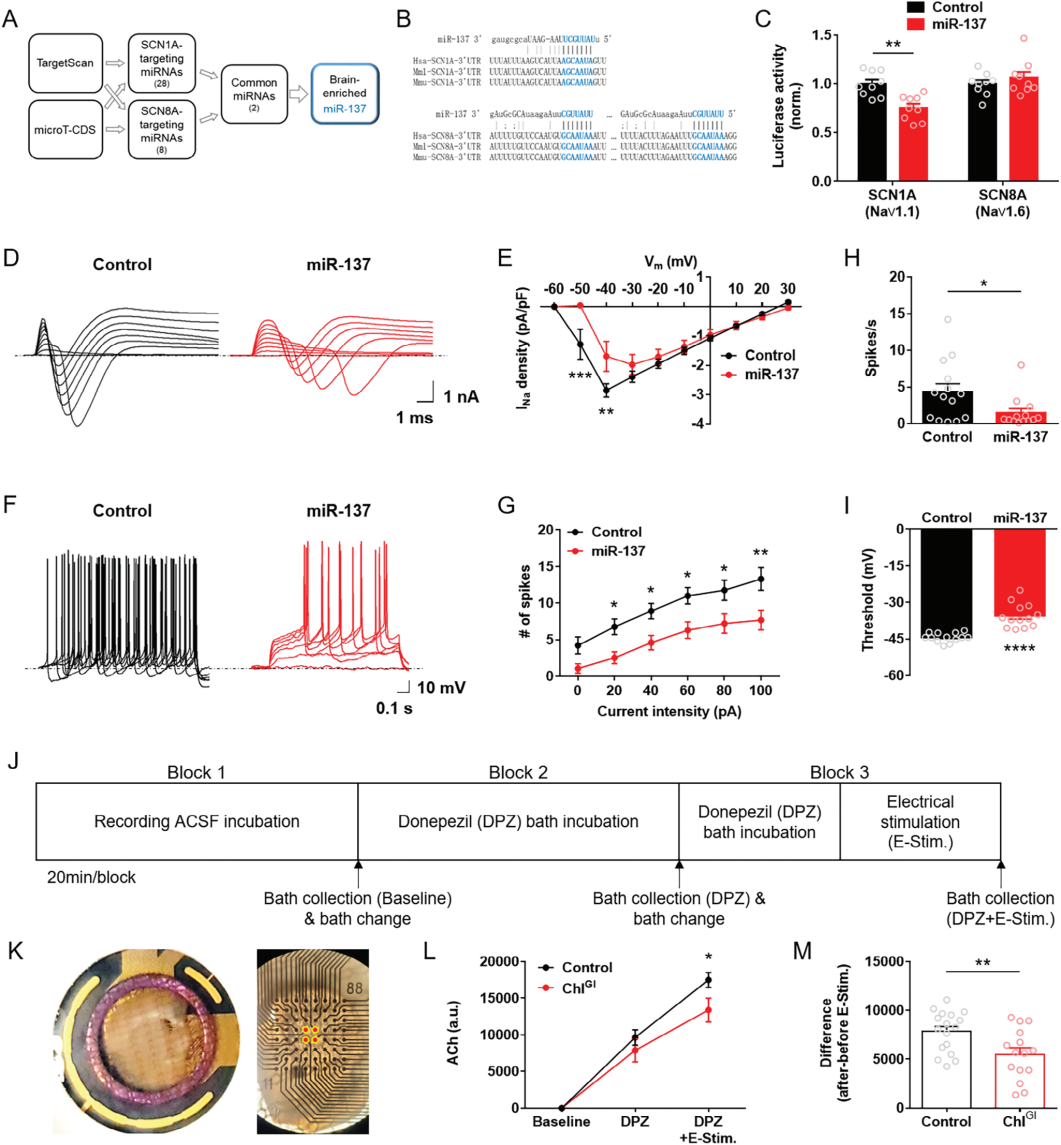
miRNA‐mediated inhibition of NaV1.1 reduces neuronal excitability in the striatal ChIs. A) Bioinformatics pipeline identifying miR‐137 as an evolutionarily conserved and brain‐enriched miRNA that can target SCN1A (Na_V_1.1) and SCN8A (Na_V_1.6). B) Seed sequence matching between miR‐137 and 3′UTR sequences in SCN1A and SCN8A mRNAs. C) Luciferase assay showing the impact of miR‐137 on the expression of SCN1A mRNA 3′UTR (*n* = 9/group) (Holm‐Sidak's *post‐hoc* test, **p = 0.0014). D) Representative traces of sodium (Na+) currents (I_Na_) in the striatal ChIs. E) Impact of miR‐137 on I_Na_ density curve. V_m_ indicates membrane potential (*n* = 13–14/group) (Holm‐Sidak's *post‐hoc* test, **p = 0.0021 and ***p = 0.0003). F) Representative traces of step current‐evoked action potentials in the striatal ChIs. G) Impact of miR‐137 on the number of electrically evoked spikes (*n* = 13–14/group) (Holm‐Sidak's *post‐hoc* test, *p = 0.0279–0.0249 and **p = 0.0047). H) Impact of miR‐137 on spontaneous firing rate (*n* = 13–14/group) (Student's *t*‐test, *p = 0.0338). I) Impact of miR‐137 on the action potential threshold (*n* = 13–14/group) (Student's *t*‐test, ****p < 0.0001). J) Multi‐electrode array (MEA) experimentation schedule for artificial cerebrospinal fluid (ACSF) bath collection at baseline, after donepezil (DPZ) treatment, and after DPZ treatment with electrical stimulation (E‐Stim.). K) A representative image of the striatal slice on MEA (left) visualized with phase contrast microscopy (right). E‐Stim. the protocol was applied to 4 electrodes (red) in the central zone. L) Acetylcholine (ACh) release from the striatal slices of Control and ChI^GI^ mice at baseline, after donepezil (DPZ) treatment, and after DPZ treatment with electrical stimulation (E‐Stim.) (*n* = 15–17/group) (Holm‐Sidak's *post‐hoc* test, *p = 0.0246). a.u., arbitrary unit. M) Differences in acetylcholine release level after versus before E‐Stim. (*n* = 15–17/group) (Student's *t*‐test, **p = 0.0084). Error bars represent SEM. See also Figures S1–S3 (Supporting Information).

We used a genetic inhibition approach in which the neuronal activity of striatal ChIs was inhibited by RNA interference‐dependent knockdown of voltage‐gated sodium channels (VGSCs), as described in previous studies,^[^ ^11^ ^]^ to investigate the role of the striatal ChIs in nicotine withdrawal. First, we sought miRNAs that could knock down the pore‐forming subunits of the VGSCs Na_V_1.1 and Na_V_1.6, which are the most abundantly expressed isoforms of the VGSC alpha subunits in the striatal ChIs.^[^ ^14^ ^]^ Bioinformatics‐based screening of the miRNAs that could inhibit Na_V_1.1 and Na_V_1.6 yielded the brain‐enriched and evolutionarily conserved miRNA miR‐137 (**Figure** **1**A,B). Next, a luciferase assay revealed that miR‐137 can inhibit the expression of SCN1A, but not SCN8A (Figure 1C) (*n* = 9/group) (Between‐subject two‐way repeated measures (RM) ANOVA, Interaction effect, *F*(1,32) = 11.23, *p* = 0.0021; Holm‐Sidak's *post‐hoc* test, **p = 0.0014). These findings demonstrated that miR‐137 could directly reduce the expression level of Na_V_1.1.

Subsequently, we designed and cloned an AAV construct that can selectively overexpress miR‐137 in a Cre‐dependent manner (Figure S1A, Supporting Information). AAV microinjection into the whole dorsal striatum of hemizygous ChAT‐Cre transgenic mice resulted in the selective expression of eGFP in the striatal ChIs (Figure S1B,C, Supporting Information), with a high penetrance rate shown by the % of ChAT+ cells per eGFP+ cells (Figure S1D, Supporting Information) (n = 3/group). In addition, immunohistochemical analysis of DAPI showed that the DAPI area was not significantly different between the Control and miR‐137 overexpression groups (Figure S1E, Supporting Information) (n = 6/group). Furthermore, immunohistochemical analysis of Na_V_1.1 showed that miR‐137 overexpression resulted in the knockdown of Na_V_1.1 expression in the striatal ChIs (Figure S1F, Supporting Information) (*n* = 6/group) (Student's *t*‐test, *t* = 3.515, *df* = 10; **p = 0.0056).

We used the patch‐clamp recording technique to verify that the genetic inhibition of striatal ChIs resulted in the functional inhibition of neuronal activity. Na_V_1.1 is a major pore‐forming alpha subunit of the VGSCs in the adult brain. The knockdown of Na_V_1.1 is expected to cause a dramatic reduction in the sodium current density. Thus, we recorded the sodium current in the striatal ChIs under miR‐137‐dependent genetic inhibition (ChI^GI^) to explore this avenue. Representative traces showed a significant reduction in the sodium current density by ChI^GI^ (Figure 1D). Quantitative comparison of the I‐V curves revealed that the sodium current density was completely abolished at a membrane potential of −50 mV and significantly reduced at −40 mV by ChI^GI^ (Figure 1E, Supporting Information) (*n* = 13–14/group) (Between‐subject two‐way RM ANOVA, Interaction effect, *F*(9225) = 4.294, *p* < 0.0001; Holm‐Sidak's *post‐hoc* test, **p = 0.0021 and ***p = 0.0003 for Control vs miR‐137). These results show that ChI^GI^ diminished the sodium current density, specifically at the range of the action potential threshold.

Subsequently, we examined the impact of ChI^GI^ on neuronal intrinsic excitability. Representative traces of the spikes generated by step current injection showed a significant decrease in the number of spikes by ChI^GI^ (Figure 1F). Quantitative comparison revealed that ChI^GI^ significantly reduced the capacity to generate action potentials (Figure 1K) (*n* = 13–14/group) (Between‐subject two‐way RM ANOVA, Group effect, *F*(1,24) = 11.28, *p* = 0.0026; Holm‐Sidak's *post‐hoc* test, *p = 0.0279–0.0249 and **p = 0.0047 for Control vs miR‐137). Moreover, ChI^GI^ also led to a significant reduction in spontaneous activity of the striatal ChIs (Figure 1H) (*n* = 13–14/group) (Student's *t*‐test, *t* = 2.245, *df* = 25; *p = 0.0338), and ChI^GI^ also caused a significant increase in the action potential threshold (Figure 1G) (*n* = 13–14/group) (Student's *t*‐test, *t* = 6.055, *df* = 24; ****p < 0.0001). Lastly, ChI^GI^ did not alter the resting membrane potential (Figure S2A, Supporting Information), but significantly reduced the cell capacitance (Figure S2B, Supporting Information) (*n* = 13–14/group) (Student's *t*‐test, *t* = 3.618, *df* = 25; **p = 0.0013).

To further verify that the genetic inhibition of striatal ChIs resulted in functional inhibition of the neuronal activity, we examined extracellular acetylcholine release in ex vivo dorsal striatal slices using a multi‐electrode array (MEA) recording system. The bathing medium was collected at 20‐min intervals from baseline, after acetylcholinesterase (AChE) inhibitor donepezil (DPZ) treatment, and after the DPZ treatment with electrical stimulation (E‐Stim.) (Figure 1J). Electrical stimulation (500 nA, fixed) was applied to the central zone of the dorsal striatum at four electrodes (Figure 1K; E‐Stim. from red electrodes). Acetylcholine (ACh) was undetectable at baseline, most likely because the dorsal striatum contains the highest level of AChE in the central nervous system^[^ ^15^ ^]^ (Figure 1L, Baseline) (*n* = 15–17/group). Interestingly, application of the AChE inhibitor donepezil unmasked the extracellular level of acetylcholine within the dorsal striatum (Figure 1L, DPZ), and electrical stimulation during donepezil incubation revealed that ChI^GI^ reduced the stimulation‐evoked acetylcholine release in the dorsal striatum (Figure 1L, DPZ+E‐Stim.) (Between‐subject two‐way RM ANOVA, Interaction effect, *F*(2,60) = 3.338, *p* = 0.0422; Holm‐Sidak's *post‐hoc* test, *p = 0.0246). Moreover, the difference in acetylcholine level before and after electrical stimulation was less prominent in the ChI^GI^ group (Figure 1M) (*n* = 15–17/group) (Student's *t*‐test, *t* = 2.824, *df* = 30; **p = 0.0084). These results collectively suggest that ChI^GI^ reduced the evoked acetylcholine release in the dorsal striatum.

On the other hand, prior studies have shown that striatal ChIs can co‐release GABA or glutamate,^[^ ^16^ ^]^ and that the striatal ChIs regulate the nigrostriatal pathway.^[^ ^17^ ^]^ Therefore, we also analyzed the extracellular release of dopamine, glutamate, and GABA from the ex vivo dorsal striatal slices using the MEA recording system. We found that ChI^GI^ reduced the extracellular release of dopamine and glutamate at baseline (Figure S3A,B, Supporting Information) (Between‐subject two‐way RM ANOVA, Interaction effect, *F*(2,60) = 4.891 and 9.174, *p* = 0.0108 and 0.0003; Holm‐Sidak's *post‐hoc* test, **p = 0.0026 for DA and ****p < 0.0001 for glutamate). In addition, we found that ChI^GI^ reduced the overall GABA release (Figure S3C, Supporting Information) (Between‐subject two‐way RM ANOVA, Group effect, *F*(1,30) = 24.56, *p* < 0.0001; Holm‐Sidak's *post‐hoc* test, ****p < 0.0001). These findings suggest that ChI^GI^ could suppress the striatal neurotransmitter release of in general.

Genetic inhibition results in highly specific and irreversible silencing of neurons, which could result in potential side effects.^[^ ^11b^ ^]^ To exclude the possibility that ChI^GI^ exerts its effect by disrupting the striatal cholinergic system, we quantified the rate‐limiting enzymes of the acetylcholine synthesis machinery. First, immunoblotting showed that the expression levels of striatal choline acetyltransferase (ChAT) and choline transporter (ChT) were unchanged by ChI^GI^ (Figure S4A,B, Supporting Information) (*n* = 4–5/group). Second, the cholinesterase assay showed that the enzymatic activity of striatal AChE was unchanged by ChI^GI^ (Figure S4C, Supporting Information) (*n* = 3/group).

Collectively, these results indicate that 1) the brain‐enriched miRNA miR‐137 can inhibit neurons by knocking down Na_V_1.1 and elevating the action potential threshold, and 2) miR‐137 gain‐of‐function in the striatal ChIs does not grossly affect the striatal acetylcholine synthesis machinery.

### 2.2 Attenuation of Somatic Signs of Nicotine Withdrawal by ChI^GI^

Next, we examined how the genetic inhibition of striatal ChIs affects the dorsal striatum‐associated behaviors. A battery of behavioral tests demonstrated that ChI^GI^ exerted no effect on sucrose preference (Figure S5A, Supporting Information) (*n* = 10–12/group), rotarod (Figure S5B, Supporting Information) (*n* = 8–9/group), nestlet shredding (Figure S5C, Supporting Information) (*n* = 9–14/group) (Within‐subject two‐way RM ANOVA, Time effect, *F*(1,21) = 63.13, *p* < 0.0001; Holm‐Sidak's *post‐hoc* test, ****p < 0.0001 for Before versus After), marble burying (Figure S5D, Supporting Information) (*n* = 9–14/group), or spatial memory (Figure S5E, Supporting Information) (*n* = 12–17/group) (Within‐subject two‐way RM ANOVA, Arm effect, *F*(1,27) = 77.40, *p* < 0.0001; Holm‐Sidak's *post‐hoc* test; Novel versus Other, ****p < 0.0001). These results are in accordance with the previous finding that striatal ChI inhibition has a subtle effect on baseline behaviors^[^ ^9a^ ^]^ (cf. effects on complex behaviors^[^ ^18^ ^]^).

**Figure 2:**
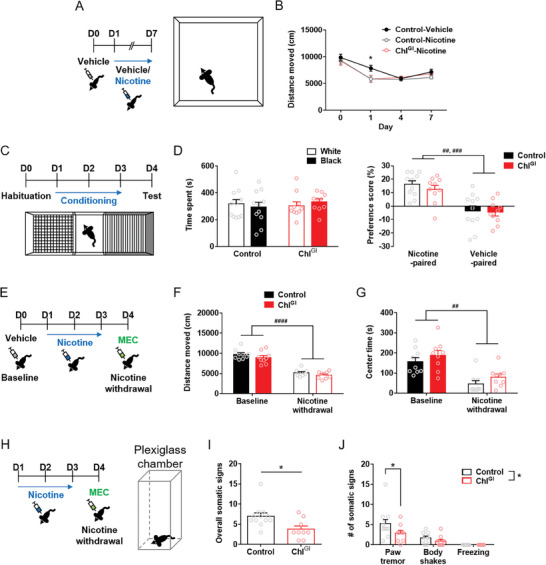
Genetic inhibition of cholinergic interneurons moderates the somatic signs of nicotine withdrawal. A) Illustrated schedule for nicotine‐induced locomotor depression test in the open field. B) Impact of ChI^GI^ on nicotine‐induced locomotor depression (*n* = 10/group) (Holm‐Sidak's *post‐hoc* test, *p = 0.0420). C) Illustrated schedule for nicotine‐conditioned place preference test. D) Impact of ChI^GI^ on the nicotine‐conditioned place preference test. (Left) Time spent in the two chambers during the pre‐test session. (Right) Preference for the two chambers during the post‐test session (*n* = 10–11/group) (Holm‐Sidak's *post‐hoc* test, ^###^ *p* = 0.0010 for Control, ^##^ *p* = 0.0030 for ChI^GI^). E) Illustrated schedule for the open field test before and during precipitated nicotine withdrawal. MEC, mecamylamine. F) Impact of ChI^GI^ on the nicotine withdrawal‐induced reduction in the distance moved in the open field (*n* = 9/group) (Holm‐Sidak's *post‐hoc* test, ^####^ *p* < 0.0001). G) Impact of ChI^GI^ on the nicotine withdrawal‐induced reduction in the time spent in the center zone in the open field test (*n* = 9/group) (Holm‐Sidak's *post‐hoc* test, ^##^ *p* < 0.0010). H) Illustrated schedule for measuring the somatic signs of nicotine withdrawal. I) Impact of ChI^GI^ on the overall somatic signs of nicotine withdrawal (*n* = 9–12/group) (Student's *t*‐test, *p = 0.0137). J) Impact of ChI^GI^ on the individual somatic signs of nicotine withdrawal (*n* = 9–12/group) (Holm‐Sidak's *post‐hoc* test, *p = 0.0214). Error bars represent SEM. See also Figures S4–S6 (Supporting Information).

Then, we examined the effects of ChI^GI^ on nicotine reward and nicotine‐induced locomotor depression. ChI^GI^ did not affect nicotine‐induced locomotor depression in the open field test (**Figure** **2**A,B) (*n* = 10/group) (Between‐subject two‐way RM ANOVA, Group effect, *F*(2,27) = 1.610, *p* = 0.2185; Holm‐Sidak's *post‐hoc* test, *p = 0.0420 for Control‐Vehicle vs other groups at Day 1). In addition, ChI^GI^ did not affect nicotine reward in the biased conditioned place preference test (Figure 2C,D; Figure S2, Supporting Information) (*n* = 10–11/group) (Figure 2D, right; Within‐subject two‐way RM ANOVA, Chamber effect, *F*(1,19) = 28.74, *p* < 0.0001; Holm‐Sidak's *post‐hoc* test, ^###^ *p* = 0.0010 for Control, ^##^ *p* = 0.0030 for ChI^GI^).

Lastly, we sought to examine the impact of ChI^GI^ on the behavioral signs of nicotine withdrawal.^[^ ^7d^ ^]^ The inhibition of striatal ChIs rescues the motor deficits present in experimental Parkinsonism,^[^ ^9^ ^]^ and the somatic symptoms of nicotine withdrawal (e.g., bodily tremor and freezing) resemble the behavioral hallmarks of Parkinson's disease.^[^ ^19^ ^]^ Therefore, we investigated whether the inhibition of striatal ChIs could alleviate the somatic signs of nicotine withdrawal. The open field test showed that ChI^GI^ did not affect the nicotine withdrawal‐induced locomotor depression (Figure 2F) (*n* = 9/group) (Within‐subject two‐way RM ANOVA, Time effect, *F*(1,16) = 292.7, *p* < 0.0001; Holm‐Sidak's *post‐hoc* test, ^####^ *p* < 0.0001 for Baseline vs Nicotine withdrawal) and anxiety‐like behavior (Figure 2G) (Within‐subject two‐way RM ANOVA, Time effect, *F*(1,16) = 36.84, *p* < 0.0001; Holm‐Sidak's *post‐hoc* test, ^##^ *p* < 0.0010 for Baseline vs Nicotine withdrawal). However, ChI^GI^ resulted in a significant attenuation of the somatic signs of nicotine withdrawal (Figure 2H,I) (*n* = 9–12/group) (Student's *t*‐test, *t* = 2.716, *df* = 19; *p = 0.0137). The breakdown into individual somatic signs revealed that ChI^GI^ markedly reduced the paw tremor induced by nicotine withdrawal (Figure 2J) (Between‐subject two‐way RM ANOVA, Group effect, *F*(1,19) = 7.375, *p = 0.0137; Holm‐Sidak's *post‐hoc* test, *p = 0.0214 for paw tremor).

A chemogenetic strategy was used to further validate that the inhibition of striatal ChIs is responsible for the reduction of somatic withdrawal signs from nicotine. First, we confirmed that clozapine N‐oxide (CNO) alone did not affect the emergence of somatic nicotine withdrawal (Figure S6A–C, Supporting Information) (*n* = 8–9/group). Next, we selectively expressed the inhibitory hM4Di DREADD receptor in the striatal ChIs (Figure S6D–F, Supporting Information). Chemogenetic inhibition of striatal ChIs (ChI‐hM4Di) resulted in a significant reduction of overall somatic signs of nicotine withdrawal (Figure S6G, Supporting Information) (*n* = 9–11/group) (Student's *t*‐test, *t* = 3.287, *df* = 18; **p = 0.0041). Moreover, an analysis of individual somatic signs showed that ChI‐hM4Di notably reduced the paw tremor (Figure S6H, Supporting Information) (Between‐subject two‐way RM ANOVA, Group effect, *F*(1,18) = 10.81, *p* = 0.0041; Holm‐Sidak's *post‐hoc* test, **p = 0.0068).

These results show that 1) the inhibition of striatal ChIs decreases the somatic signs of nicotine withdrawal, and 2) the inhibition of striatal ChIs does not affect nicotine reward, nicotine‐induced locomotor depression, and striatum‐associated behaviors.

### 2.3 Inhibition of Nicotine‐Dependent Changes in the Striatal Neural Population Spikes by ChI^GI^

**Figure 3:**
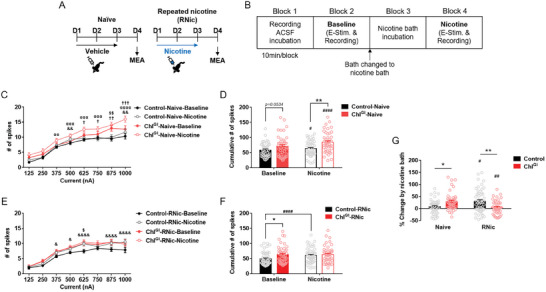
ChI^GI^ counteracts the neural activity responses to nicotine in the dorsal striatum. A) Illustrated schedule for multi‐electrode array (MEA) recording. B) MEA recordings were conducted before and after bath incubation of nicotine. E‐Stim., electrical stimulation. C) Current‐evoked neural population spikes in the dorsal striatum of nicotine‐naïve mice (*n* = 44–46/group) (Holm‐Sidak's *post‐hoc* test; compared groups and exact *p*‐values are summarized in Table S1, Supporting Information). Control, AAV‐eGFP injection into the dorsal striatum; Naïve, in vivo saline exposure; RNic, in vivo repeated nicotine exposure; Baseline, *ex vivo* ACSF application; Nicotine, *ex vivo* nicotine application. D) The cumulative number of current‐evoked neural population spikes in the dorsal striatum of nicotine‐naïve mice (*n* = 44–46/group) (Holm‐Sidak's *post‐hoc* test, **p = 0.0018, ^#^ *p* = 0.0492, ^####^ *p* < 0.0001). E) Current‐evoked neural population spikes in the dorsal striatum of mice after RNic (*n* = 44–53/group) (Holm‐Sidak's *post‐hoc* test; compared groups and exact *p*‐values are summarized in Table S1, Supporting Information). F) The cumulative number of current‐evoked neural population spikes in the dorsal striatum of nicotine‐experienced mice (*n* = 44–53/group) (Holm‐Sidak's *post‐hoc* test, *p = 0.0192, ^####^ *p* < 0.0001). G) The percentage (%) of changes in neural activity by bath nicotine application (*n* = 44–53/group) (Holm‐Sidak's *post‐hoc* test, *p = 0.0193, **p = 0.0051, ^#^ *p* = 0.0111, ^##^ *p* = 0.0088). Error bars represent SEM. Statistical significance for (C) and (E) are summarized in Table S1 (Supporting Information). See also Figure S7 (Supporting Information).

**Figure 4:**
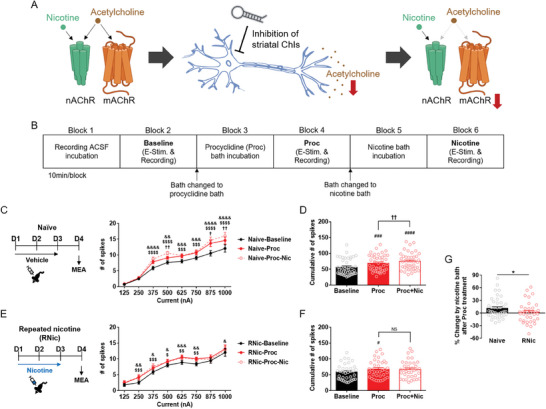
Clinically‐approved muscarinic antagonist procyclidine mimics the electrophysiological effect of ChI^GI^. A) Illustrated model for mAChR‐dependent mechanism of action by ChI^GI^. In the control state (left), nicotine binds to and activates nAChR, while acetylcholine binds to and activates both nAChR and mAChR. After ChI^GI^ (middle), striatal acetylcholine release is reduced. During nicotine exposure, ChI^GI^ leads to a reduction in mAChR signaling but not nAChR (right). B) MEA recording conducted before and after bath incubation of the broad‐spectrum muscarinic antagonist procyclidine (Proc) in wild‐type mice. C) Current‐evoked neural population spikes in the dorsal striatum of nicotine‐naïve mice at baseline, after Proc treatment, and after Proc+nicotine (Nic) treatment (*n* = 42) (Holm‐Sidak's *post‐hoc* test; compared groups and exact *p*‐values are summarized in Table S1, Supporting Information). D) The cumulative number of current‐evoked neural population spikes in the dorsal striatum of nicotine‐naïve mice at baseline, after Proc treatment, and after Proc+Nic treatment (*n* = 42) (Holm‐Sidak's *post‐hoc* test, ^###^ *p* = 0.0002, ^####^ *p* < 0.0001, ^††^ *p* = 0.0073). E) Current‐evoked neural population spikes in the dorsal striatum of mice after repeated nicotine exposure at baseline, after Proc treatment, and after Proc+Nic treatment (*n* = 36) (Holm‐Sidak's *post‐hoc* test; compared groups and exact *p*‐values are summarized in Table S1, Supporting Information). F) The cumulative number of current‐evoked neural population spikes in the dorsal striatum of mice after repeated nicotine exposure at baseline, after Proc treatment, and after Proc+Nic treatment (*n* = 36) (Holm‐Sidak's *post‐hoc* test, ^#^ *p* = 0.0168). NS, not significant. G) The percentage (%) of changes in neural activity (*n* = 36–42/group) (Student's *t*‐test, *p = 0.0390). Error bars represent SEM. Statistical significance for (C) and (E) are summarized in Table S1 (Supporting Information). See also Figure S8 (Supporting Information).

Neural population spikes are generated by the extracellular summation of neural firings in a local brain network,^[^ ^20^ ^]^ which is directly correlated with the synchronized neuronal activity of a brain region. Studies have shown that dysfunctional changes in the stochastic probability to evoke population spikes are associated with brain disorders.^[^ ^21^ ^]^ As such, we analyzed how ChI^GI^ affects nicotine‐induced alterations in the neural population spikes in the dorsal striatum through ex vivo MEA recording (Figure S2, Supporting Information for general setup and protocol of the MEA recording; see Table S1 (Supporting Information) for the compilation of statistical significance from the comparison of the MEA data per step current in Figures 3C,E and 4C,E).

Evoked neural population spikes were measured in the dorsal striatum of the control (AAV‐eGFP) or ChI^GI^ (genetic inhibition of striatal cholinergic interneurons) mice after in vivo repeated exposure to either vehicle (Naïve group; nicotine‐naïve condition) or nicotine (RNic group; nicotine‐primed condition) (**Figure** **3**A), and sequentially in ex vivo drug‐free (baseline) and drug‐applied (nicotine) conditions (Figure 3B).

In the investigation of the ex vivo nicotine effect in the nicotine‐naïve mice (Figure 3C,D) (*n* = 44–46/group), the control mice showed little increase in the number of striatal neural population spikes in response to the ex vivo nicotine treatment, but ChI^GI^ mice showed a dramatic increase in the number of striatal neural population spikes in response to the ex vivo nicotine (Figure 3D) (Within‐subject two‐way RM ANOVA, Interaction effect, *F*(1,88) = 8.167*, p =* 0.0053; Holm‐Sidak's *post‐hoc* test, ^#^ *p* = 0.0492 for Control‐Naïve‐Baseline versus Control‐Naïve‐Nicotine, ^####^ *p* < 0.0001 for ChI^GI^‐Naïve‐Baseline versus ChI^GI^‐Naïve‐Nicotine). In the investigation of the *ex vivo* ChI^GI^ effect in the nicotine‐naïve mice, ChI^GI^ resulted in an increased number of striatal neural population spikes compared to the control (Figure 3D) (Between‐subject two‐way RM ANOVA, Interaction effect, *F*(1,88) = 8.167*, p =* 0.0053; Holm‐Sidak's *post‐hoc* test, *p* = 0.0534 for Control‐Naïve‐Baseline vs ChI^GI^‐Naïve‐Baseline, **p = 0.0018 for Control‐Naïve‐Nicotine vs ChI^GI^‐Naïve‐Nicotine).

In contrast, in the investigation of the ex vivo nicotine effect in the nicotine‐primed mice (Figure 3E,F) (*n* = 44–53/group), the control showed a significant increase in the number of striatal neural population spikes in response to the ex vivo nicotine treatment, but ChI^GI^ did not show any change in the number of striatal neural population spikes in response to the ex vivo nicotine (Figure 3F) (Within‐subject two‐way RM ANOVA, Interaction effect, *F*(1,95) = 8.299, *p* = 0.0049; Holm‐Sidak's *post‐hoc* test, ^####^ *p* < 0.0001 for Control‐RNic‐Baseline vs Control‐RNic‐Nicotine). In the investigation of the *ex vivo* ChI^GI^ effect in the nicotine‐primed mice, ChI^GI^ resulted in an increased number of striatal neural population spikes compared to the control (Figure 3F) (Between‐subject two‐way RM ANOVA, Interaction effect, *F*(1,95) = 8.299, *p* = 0.0049; Holm‐Sidak's *post‐hoc* test, *p = 0.0192 for Control‐RNic‐Baseline vs ChI^GI^‐RNic‐Baseline).

Further analysis of the ex vivo nicotine‐induced percentage changes in the striatal neural population spikes revealed that, in the nicotine‐naïve mice, the control showed no change in the number of striatal neural population spikes by the ex vivo nicotine; however, ChI^GI^ caused an ex vivo nicotine‐induced enhancement of the striatal neural population spikes (Figure 3G, Naïve) (Two‐way RM ANOVA, Interaction effect, *F*(1184) = 18.82, *p* < 0.0001; Holm‐Sidak's *post‐hoc* test, *p = 0.0193 for Control‐Naïve vs ChI^GI^‐Naïve). Conversely, in the nicotine‐primed mice, the control showed an ex vivo nicotine‐induced increase in the number of striatal neural population spikes, but ChI^GI^ blocked the *ex vivo* nicotine‐induced change in the striatal neural population spikes (Figure 3G, RNic) (Holm‐Sidak's *post‐hoc* test, Control‐RNic vs ChI^GI^‐RNic, **p = 0.0051). Regarding the nicotine effect, the Control showed an ex vivo nicotine‐induced increase in the number of striatal population spikes only after the in vivo nicotine priming (Figure 3G, Control) (Holm‐Sidak's *post‐hoc* test, Control‐Naïve vs Control‐RNic, ^#^ *p* = 0.0111); however, ChI^GI^ showed a nicotine‐induced increase in the number of striatal population spikes only in the in vivo nicotine‐naïve condition (Figure 3G, ChI^GI^) (Holm‐Sidak's *post‐hoc* test, ChI^GI^‐Naïve vs ChI^GI^‐RNic, ^##^ *p* = 0.0088).

Collectively, these results show that 1) the ex vivo nicotine does not significantly affect the striatal neural population spikes in the nicotine‐naïve striatum, but ChI^GI^ renders the nicotine‐naïve striatum sensitive to nicotine; 2) the ex vivo nicotine significantly enhances the striatal neural population spikes in the nicotine‐primed striatum, but ChI^GI^ blocks the electrophysiological effect of the nicotine on the nicotine‐primed striatum; 3) inhibition of striatal ChIs generally increases the number of striatal neural population spikes.

### 2.4 Attenuation of Nicotine‐Dependent Changes in Striatal Population Spikes by Antimuscarinic Treatment

The striatal ChIs release acetylcholine, which activates nAChRs and mAChRs to exert diverse neurobiological effects. In normal conditions, both nAChRs and mAChRs are readily activated by acetylcholine release and nicotine application (**Figure** **4**A, left), but ChI^GI^ would render the striatal ChIs to release a lower amount of acetylcholine (Figure 4A, middle). In such a condition, the nAChRs would be activated by the exogenous nicotine, but mAChR signaling would be comparatively reduced (Figure 4A, right). Through this model, we hypothesized that the ChI^GI^‐mediated ectopic reduction of muscarinic receptor signaling is responsible for the ChI^GI^‐dependent inhibition of nicotine‐induced electrophysiological effects in the dorsal striatum.

To explore this avenue, we applied a broad‐spectrum, centrally‐acting, clinically‐approved muscarinic antagonist procyclidine (Proc) to the dorsal striatum during the MEA recording (Figure 4B). In the nicotine‐naïve striatum (Naïve), as with the ChI^GI^, the ex vivo procyclidine increased the number of striatal neural population spikes (Figure 4C,D) (*n* = 42) (in Figure 4D, Within‐subject one‐way RM ANOVA, *F*(1.507,61.80) = 21.77, *p* < 0.0001; Holm‐Sidak's *post‐hoc* test, ^###^ *p* = 0.0002, Baseline vs Proc). More importantly, as with the ChI^GI^, the ex vivo procyclidine treatment caused the *ex vivo* nicotine (Nic) to increase the number of striatal population spikes in the nicotine‐naïve striatum (Figure 4D) (Holm‐Sidak's *post‐hoc* test, ^####^ *p* < 0.0001 for Baseline vs Proc+Nic, ^††^ *p* = 0.0073 for Proc vs Proc+Nic).

In contrast, in the nicotine‐primed striatum (RNic), the *ex vivo* procyclidine again increased the number of striatal population spikes (Figure 4E,F) (*n* = 36) (in Figure 4F, Within‐subject one‐way RM ANOVA, *F*(1.620,56.70) = 5.346, *p* = 0.0115; Holm‐Sidak's *post‐hoc* test, ^#^ *p* = 0.0168 for Baseline vs Proc), but the *ex vivo* procyclidine abolished the ex vivo nicotine‐dependent increase in the striatal population spikes in the nicotine‐primed striatum as with the ChI^GI^ (Figure 4F) (Holm‐Sidak's *post‐hoc* test, not significant (NS) for Proc vs Proc+Nic).

Analysis of the ex vivo nicotine‐induced percent changes in the number of striatal population spikes after the ex vivo procyclidine treatment showed that, as with the ChI^GI^, the ex vivo procyclidine induced the *ex vivo* nicotine‐dependent increase in the striatal population spikes in the nicotine‐naïve striatum; however, procyclidine blocked the ex vivo nicotine‐dependent increase in striatal population spikes in the nicotine‐primed striatum (Figure 4G) (Student's *t*‐test, *t* = 1.787, *df* = 76; *p = 0.0390).

Additionally, we examined the electrophysiological effect of the antinicotinic drug, mecamylamine (MEC) (Figure S8A,B, Supporting Information), which mimics the in vivo protocol for mecamylamine‐induced precipitation of nicotine withdrawal. We found that the ex vivo mecamylamine affected neither the basal number of striatal population spikes nor the ex vivo nicotine‐dependent increase in the number of striatal population spikes (Figure S8C,D, Supporting Information) (*n* = 44–47/group).

Furthermore, we also examined the electrophysiological effect of a muscarinic agonist, oxotremorine‐M (OxoM) (Figure S8E, Supporting Information) to examine whether muscarinic agonism could reverse the electrophysiological effects of ChI^GI^. We found that the ex vivo oxotremorine‐M treatment increased the number of striatal population spikes in the nicotine‐primed striatum (Figure S8F,G, Supporting Information) (*n* = 40/group) (in Figure S8G (Supporting Information), Within‐subject one‐way RM ANOVA, *F*(1.775,69.22) = 27.86, *p* < 0.0001; Holm‐Sidak's *post‐hoc* test, ^####^ *p* < 0.0001 for Baseline vs other groups); however, the ex vivo oxotremorine‐M was unable to reverse the ChI^GI^‐dependent blockade of the ex vivo nicotine‐induced increase in the striatal population spikes in the nicotine‐primed striatum (Holm‐Sidak's *post‐hoc* test, not significant for OxoM vs OxoM+Nic).

In summary, these results show that 1) the antimuscarinic drug procyclidine can mimic the therapeutic electrophysiological effects of the striatal ChI inhibition in the nicotine‐primed striatum, 2) the antinicotinic drug mecamylamine has no electrophysiological effect on the nicotine‐primed striatum, and 3) the muscarinic agonist oxotremorine‐M does not affect the ex vivo nicotine‐induced increase in the striatal population spikes in the nicotine‐primed striatum after striatal ChI inhibition.

### 2.5 Inhibition of the Nicotine Withdrawal‐Induced Decrease in Striatal Dopamine Release by ChI^GI^ and Procyclidine

**Figure 5:**
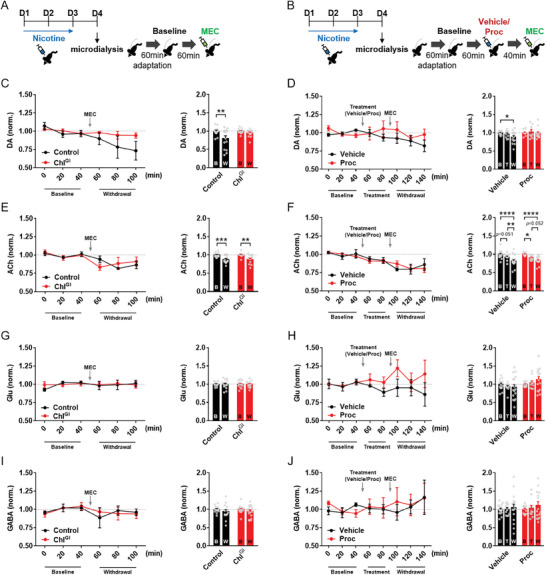
ChI^GI^ and procyclidine prevent the nicotine withdrawal‐dependent reduction in striatal dopamine release. A) Illustrated schedule for microdialysis experiment after ChI^GI^. MEC, mecamylamine. B) Illustrated schedule for microdialysis experiment with procyclidine (Proc) administration. C) (Left) Striatal dopamine (DA) release in Control and ChI^GI^ mice before and after precipitation of nicotine withdrawal with MEC. norm., normalized to the Baseline level. (Right) Summation of striatal DA release during nicotine withdrawal in Control and ChI^GI^ mice (*n* = 4–6/group) (Holm‐Sidak's *post‐hoc* test, **p = 0.0011). B, Baseline; W, Withdrawal. D) (Left) Striatal DA release in nicotine‐withdrawn mice after vehicle or procyclidine treatment. (Right) Summation of striatal DA release during nicotine withdrawal in vehicle‐treated and procyclidine‐treated mice (*n* = 4–6/group) (Holm‐Sidak's *post‐hoc* test, *p = 0.0274). B, Baseline; T, Treatment (Vehicle/Proc); W, Withdrawal. E) (Left) Striatal acetylcholine (ACh) release in Control and ChI^GI^ mice before and after precipitation of nicotine withdrawal with mecamylamine (MEC). (Right) Summation of striatal ACh release in Control and ChI^GI^ mice (*n* = 4–6/group) (Holm‐Sidak's *post‐hoc* test, ***p = 0.0006, **p = 0.0012). F) (Left) Striatal ACh release in nicotine‐withdrawn mice after vehicle or procyclidine treatment. (Right) Summation of striatal ACh release after Vehicle/Proc treatment and after MEC injection (*n* = 4–6/group) (Holm‐Sidak's *post‐hoc* test, **p = 0.0075, *p = 0.0462). G) (Left) Striatal glutamate (Glu) release in Control and ChI^GI^ mice before and after precipitation of nicotine withdrawal with MEC. (Right) Summation of striatal Glu release after nicotine withdrawal. H) (Left) Striatal Glu release in nicotine‐withdrawn mice after vehicle or procyclidine treatment. (Right) Summation of striatal Glu release after nicotine withdrawal in vehicle‐treated and procyclidine‐treated mice. I) (Left) Striatal GABA release in Control and ChI^GI^ mice before and after precipitation of nicotine withdrawal with MEC. (Right) Summation of striatal GABA release after nicotine withdrawal. J) (Left) Striatal GABA release in nicotine‐withdrawn mice after vehicle or procyclidine treatment. (Right) Summation of striatal GABA release after nicotine withdrawal in vehicle‐treated and procyclidine‐treated mice. Error bars represent SEM. See also Figures S9 (Supporting Information).

Striatal ChIs strongly gate striatal dopamine release,^[^ ^1c^ ^]^ and nicotine modulates this striatal dopamine release in a complex manner.^[^ ^22^ ^]^ Here, we subsequently investigated how ChI^GI^ and the muscarinic antagonist, procyclidine, affect the nicotine withdrawal‐induced changes in striatal neurotransmitter release in vivo after repeated nicotine exposure (**Figure** **5**A,B, respectively for ChI^GI^ and procyclidine; Figure S9A (Supporting Information) for the cannulation site) (*n* = 4–6/group).

We first found that nicotine withdrawal significantly reduced the striatal dopamine release (Figure 5C,D) (in Figure 5C right, Within‐subject two‐way RM ANOVA, Treatment effect, *F*(1,62) = 10.80, *p* = 0.0017; Holm‐Sidak's *post‐hoc* test, **p = 0.0011 for Control‐B vs Control‐W) (in Figure 5D right, Within‐subject two‐way RM ANOVA, Treatment effect, *F*(2,66) = 2.478, *p* = 0.0917; Holm‐Sidak's *post‐hoc* test, *p = 0.0274 for Vehicle‐B vs Vehicle‐W). Importantly, both ChI^GI^ and procyclidine blocked the nicotine withdrawal‐dependent reduction in striatal dopamine release (in Figure 5C, ChI^GI^‐B vs ChI^GI^‐W) (in Figure 5D, Proc‐B vs Proc‐W).

Second, we also found that nicotine withdrawal seemed to reduce striatal acetylcholine release significantly (Figure 5E) (in Figure 5E right, Within‐subject two‐way RM ANOVA, Treatment effect, *F*(1,50) = 26.68, *p* < 0.0001; Holm‐Sidak's *post‐hoc* test, ***p = 0.0006 for Control‐B vs Control‐W, **p = 0.0012 for ChI^GI^‐B vs ChI^GI^‐W). However, we found that the intraperitoneal injection of any agent caused a significant decrease in the striatal acetylcholine release (Figure 5F) (in Figure 5F right, Within‐subject two‐way RM ANOVA, Treatment effect, F(2,82) = 26.53, *p* < 0.0001; Holm‐Sidak's *post‐hoc* test, *p* = 0.0510 for Vehicle‐B versus Vehicle‐T, **p = 0.0075 for Vehicle‐T versus Vehicle‐W, *p = 0.0462 for Proc‐B vs Proc‐T, *p* = 0.0516 for Proc‐T vs Proc‐W).

Previous studies have shown that a subpopulation of striatal ChIs co‐releases GABA or glutamate^[^ ^16^ ^]^ and that the striatal ChIs also control GABA release from the nigrostriatal input.^[^ ^17^ ^]^ Thus, we additionally examined how nicotine withdrawal, ChI^GI^, and procyclidine affect the striatal glutamate or GABA release. We found that nicotine withdrawal did not affect the striatal glutamate or GABA release and that both ChI^GI^ and procyclidine did not alter the striatal glutamate or GABA release during nicotine withdrawal (Figure 5G–J).

Collectively, these results indicate that 1) striatal ChI inhibition or muscarinic antagonist can prevent the nicotine withdrawal‐induced reduction in striatal dopamine release, 2) striatal acetylcholine release is reduced in response to the experience of intraperitoneal injection, and 3) striatal glutamate/GABA release is unaffected by nicotine withdrawal, striatal ChI inhibition, or muscarinic antagonism.

### 2.6 Attenuation of Physical Signs of Nicotine Withdrawal by Antimuscarinic Treatment

**Figure 6:**
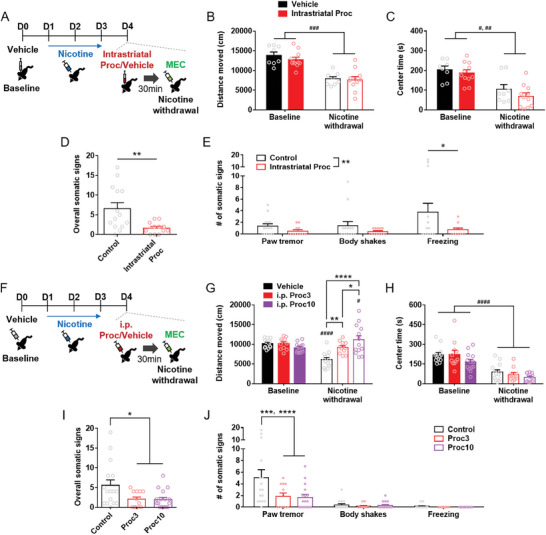
Procyclidine treatment ameliorates the physical signs of nicotine withdrawal. A) Illustrated schedule for the open field test and measurement of the somatic signs of nicotine withdrawal after intrastriatal administration of procyclidine (Proc). MEC, mecamylamine. B) Impact of intrastriatal procyclidine on the nicotine withdrawal‐induced locomotor depression (*n* = 8–11/group) Holm‐Sidak's *post‐hoc* test, ^###^ *p* = 0.0002). C) Impact of intrastriatal procyclidine on the nicotine withdrawal‐induced reduction in the time spent in the center zone of the open field test (*n* = 8–11/group) (Holm‐Sidak's *post‐hoc* test, ^#^ *p* = 0.0100, ^##^ *p* = 0.0019). D) Impact of intrastriatal procyclidine on the overall somatic signs of nicotine withdrawal (*n* = 12–14/group) (Student's *t*‐test, **p = 0.0066). E) Impact of intrastriatal procyclidine on the individual somatic signs of nicotine withdrawal (*n* = 12–14/group) (Holm‐Sidak's *post‐hoc* test, *p = 0.0210). F) Illustrated schedule for the open field test and measurement of the somatic signs of nicotine withdrawal after systemic administration of Proc. G) Impact of systemic procyclidine on the nicotine withdrawal‐induced locomotor depression (*n* = 11–13/group) (Holm‐Sidak's *post‐hoc* test, ^#^ *p* = 0.0355, ^####^ *p* < 0.0001, *p = 0.0258, **p = 0.0011, ****p < 0.0001). Proc3, systemic administration of 3.0 mg k^−1^g procyclidine; Proc10, systemic administration of 10.0 mg k^−1^g procyclidine. H) Impact of systemic procyclidine on the nicotine withdrawal‐induced reduction in the time spent in the center zone of the open field test (*n* = 11–13/group) (Holm‐Sidak's *post‐hoc* test, ^####^ *p* < 0.0001). I) Impact of systemic procyclidine on the overall somatic signs of nicotine withdrawal (*n* = 12–17/group) (Holm‐Sidak's *post‐hoc* test, *p = 0.0246–0.0336). J) Impact of systemic procyclidine on the individual somatic signs of nicotine withdrawal (*n* = 12–17/group) (Holm‐Sidak's *post‐hoc* test, ***p = 0.0003, ****p < 0.0001). Error bars represent SEM. See also Figure S9 (Supporting Information).

We found that the clinically‐approved antimuscarinic drug procyclidine mimicked the therapeutic effects of ChI^GI^ on striatal electrophysiology and neurotransmission. Therefore, we subsequently explored whether in vivo procyclidine treatment could ameliorate the physical signs of nicotine withdrawal. First, we examined whether the dorsal striatum‐specific administration of procyclidine can ameliorate the physical signs of nicotine withdrawal (**Figure** **6**A; Figure S9B, Supporting Information for the cannulation site). The intrastriatal administration of procyclidine did not prevent locomotor depression or anxiety‐like behavior produced by nicotine withdrawal (Figure 6B,C) (*n* = 8–11/group) (for Figure 6B, Within‐subject two‐way RM ANOVA, Treatment effect, *F*(1,17) = 49.30, *p* < 0.0001; Holm‐Sidak's *post‐hoc* test, ^###^ *p* = 0.0002 for Baseline versus Nicotine withdrawal) (for Figure 6C, Within‐subject two‐way RM ANOVA, Treatment effect, *F*(1,17) = 22.91, *p* = 0.0002; Holm‐Sidak's *post‐hoc* test, ^#^ *p* = 0.0100 for Vehicle‐Baseline versus Vehicle‐Nicotine withdrawal, ^##^ *p* = 0.0019 for Intrastriatal Proc‐Baseline versus Intrastriatal Proc‐Nicotine withdrawal). However, as with ChI^GI^, intrastriatal procyclidine could reduce the overall somatic signs of nicotine withdrawal (Figure 6D) (*n* = 12–14/group) (Student's *t*‐test, *t* = 2.973, *df* = 24; **p = 0.0066). Unexpectedly, analysis of the individual somatic signs revealed that the mice, after intrastriatal administration, exhibited an increase in freezing behavior during nicotine withdrawal, but the somatic signs of nicotine withdrawal including freezing were fully ameliorated by the intrastriatal procyclidine treatment (Figure 6E) (Between‐subject two‐way RM ANOVA, Group effect, *F*(1,24) = 8.838, **p = 0.0066; Holm‐Sidak's *post‐hoc* test, *p = 0.0210 for freezing).

Next, we examined whether the systemic administration of procyclidine could attenuate the physical signs of nicotine withdrawal (Figure 6F). Nicotine withdrawal again reduced the locomotor activity in the open field (Figure 6G) (*n* = 11–13/group) (Within‐subject two‐way RM ANOVA, Treatment effect, *F*(1,34) = 4.645, *p* = 0.0383; Holm‐Sidak's *post‐hoc* test, ^####^ *p* < 0.0001 for Vehicle‐Baseline vs Vehicle‐Nicotine withdrawal). Interestingly, the systemic administration of procyclidine dose‐dependently reversed the nicotine withdrawal‐induced locomotor depression (Figure 6G) (*n* = 11–13/group) (Between‐subject two‐way RM ANOVA, Group effect, *F*(2,34) = 6.311, *p* = 0.0047; Holm‐Sidak's *post‐hoc* test, **p = 0.0011 for Vehicle‐Nicotine withdrawal versus i.p. Proc3‐Nicotine withdrawal, ****p < 0.0001 for Vehicle‐Nicotine withdrawal versus i.p. Proc10‐Nicotine withdrawal, *p = 0.0258 for i.p. Proc3‐Nicotine withdrawal versus i.p. Proc10‐Nicotine withdrawal, ^#^ *p* = 0.0355 for i.p. Proc10‐Baseline versus i.p. Proc10‐Nicotine withdrawal). Alternatively, the systemic administration of procyclidine did not affect the anxiety‐like behavior from nicotine withdrawal (Figure 6H) (Within‐subject two‐way RM ANOVA, Treatment effect, *F*(1,34) = 96.00, *p* < 0.0001; Holm‐Sidak's *post‐hoc* test, ^####^ *p* < 0.0001 for Baseline vs Nicotine withdrawal).

In addition, the systemic administration of procyclidine was able to attenuate the overall somatic signs of nicotine withdrawal, irrespective of the dose (Figure 6I) (*n* = 12–17/group) (Between‐subject one‐way ANOVA, *F*(2,42) = 4.742, *p* = 0.0139; Holm‐Sidak's *post‐hoc* test; Control vs Proc3, *p = 0.0336; Control vs Proc10, *p = 0.0246). Analysis of the individual somatic signs of nicotine withdrawal showed that paw tremor was significantly reduced by the systemic procyclidine treatment (Figure 6J) (Between‐subject two‐way RM ANOVA, Group effect, *F*(2,42) = 4.742, *p* = 0.0139; Holm‐Sidak's *post‐hoc* test, ***p = 0.0003 for Control vs Proc3, ****p < 0.0001 for Control vs Proc10).

These results show that 1) procyclidine does not affect the nicotine withdrawal‐induced increase in anxiety‐like behavior, 2) intrastriatal or systemic procyclidine reduces the somatic signs of nicotine withdrawal, and 3) systemic procyclidine treatment also ameliorates the nicotine withdrawal‐induced locomotor depression.

## 3 Discussion

**Figure 7:**
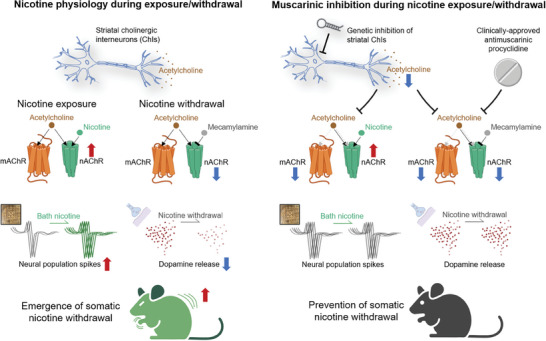
Graphical summary of the role of striatal cholinergic interneurons in physical nicotine withdrawal.

This study presented evidence that the inhibition of striatal cholinergic interneurons or the clinically approved antimuscarinic drug procyclidine could reduce the physical signs of nicotine withdrawal, block the ex vivo nicotine‐induced changes in the striatal neural population spikes, and prevent the nicotine withdrawal‐induced decrease in striatal dopamine release (**Figure** **7**). Additionally, it showed that the brain‐enriched miRNA miR‐137 could inhibit the neuronal activity by knocking down a voltage‐gated sodium channel subunit and that this inhibition of striatal cholinergic interneurons had little effect on striatum‐associated behaviors or nicotine withdrawal‐induced anxiety behavior. These results have established a connection between the striatal cholinergic system and nicotine dependence. Notably, the drug repurposing approach has revealed that the clinically‐approved muscarinic antagonist procyclidine has potential as a readily translatable therapeutic against nicotine withdrawal syndrome.

### 3.1 The Mouse Model of Nicotine Dependence

Mounting evidence to date has validated that withdrawal symptoms develop with nondaily, repeated, or even a single experience of the drug/medication.^[^ ^23^ ^]^ Multiple independent studies have shown that short‐term nicotine exposure during the early experimentation stage can provoke behavioral signs of withdrawal in both rodents^[^ ^23^, ^24^ ^]^ and humans.^[^ ^12^, ^25^ ^]^ Such an early manifestation of withdrawal signs indicates that an initiating stage exists and is essential in developing physical dependence,^[^ ^26^ ^]^ previously termed acute dependence.^[^ ^23^, ^24^, ^26^ ^]^ Following the line of these studies, here we used a mouse model of nicotine dependence in which the mice underwent repeated nicotine exposure followed by mecamylamine‐induced precipitation of nicotine withdrawal.^[^ ^7d^ ^]^ An earlier study reported that the physical aspect of nicotine withdrawal (locomotor depression and somatic signs) was most prominent in a mouse model of acute dependence and that the magnitude of the physical withdrawal signs was comparable to those reported in prior seminal studies implementing the chronic nicotine exposure protocol.^[^ ^7^, ^27^ ^]^ Indeed, our data show that nicotine dependence was marked by a steep increase in physical signs, thereby replicating and supporting the previous findings.

### 3.2 The Neural Correlates of Physical Nicotine Withdrawal

Established in pioneering preclinical studies^[^ ^7^, ^27^ ^]^ and clinical studies,^[^ ^28^ ^]^ paw/hand tremor is the single most prominent somatic sign of nicotine withdrawal. Indeed, we found that paw tremor was most notably increased in the mouse model of nicotine withdrawal. In addition, we found that inhibition of striatal cholinergic interneurons significantly reduced the nicotine withdrawal‐induced paw tremor. These findings suggest that the nicotine withdrawal‐induced paw tremor could be due to a disruption in either 1) central neuronal pacemakers or 2) oscillations in the neural circuit feedforward/feedback loop, as hypothesized previously.^[^ ^29^ ^]^ Considering that an autonomous pacemaker mechanism characterizes the striatal cholinergic interneurons,^[^ ^30^ ^]^ the pacemaking activity of the striatal cholinergic interneurons may be directly correlated with the nicotine withdrawal‐induced paw tremor. Alternatively, the oscillatory activity of the cortico‐striatal‐thalamic loop could be affected by striatal cholinergic interneurons,^[^ ^31^ ^]^ which may also contribute to the manifestation of nicotine withdrawal‐induced paw tremor. Further studies are required to identify the correlation between the neuronal activity of the striatal cholinergic interneurons and paw tremors.

Outside of the dorsal striatum, the physical symptoms of nicotine withdrawal have been primarily associated with the habenulo‐interpeduncular circuit.^[^ ^32^ ^]^ This study presented evidence on the dorsal striatum as another brain correlate of physical nicotine withdrawal, as previously implied.^[^ ^33^ ^]^ A direct neural connection has yet to be identified between the habenulo‐interpeduncular circuit and the dorsal striatum. However, neural circuit studies have hinted that the interpeduncular nucleus sends indirect input to the dorsal striatum through the brainstem, via the laterodorsal tegmental nucleus and dorsal raphe nucleus.^[^ ^34^ ^]^ Whereas the serotonergic inputs from the dorsal raphe nucleus have been implicated in the pathophysiology of nicotine withdrawal,^[^ ^35^ ^]^ the association between the laterodorsal tegmental nucleus and nicotine withdrawal has not been explored to date. To summarize, these studies suggest that future work should focus on how the information relayed within the habenulo‐interpeduncular‐brainstem‐striatum circuit regulates physical nicotine withdrawal.

### 3.3 Muscarinic Receptor Signaling in Nicotine Withdrawal

Our data revealed that the striatal cholinergic interneurons control the somatic signs of nicotine withdrawal through muscarinic acetylcholine receptor (mAChR) signaling. This finding suggests that the nicotine‐dependent activation of nicotinic acetylcholine receptors (nAChRs) and its interaction with muscarinic signaling may be an important phenomenon in the pathophysiology of nicotine withdrawal. Few studies have investigated the receptor‐receptor interaction between nAChRs and mAChRs. nAChR‐dependent depolarization is required to activate the co‐localized mAChRs.^[^ ^36^ ^]^ Nicotine withdrawal leads to desensitization of the nAChRs,^[^ ^37^ ^]^ which could decrease the activity of the mAChRs by reducing the depolarization of the co‐localized nAChRs. However, the nicotine withdrawal‐induced desensitization of the nAChRs could also increase the sensitivity of the mAChRs^[^ ^38^ ^]^ by enhancing the receptor availability of the mAChRs for acetylcholine binding. Follow‐up studies are warranted to clarify the nAChR‐mAChR interaction and its role in nicotine withdrawal, and vice versa.

Additionally, we unexpectedly found a U‐shaped curve relationship between the muscarinic receptor signaling and striatal neural activity because both muscarinic antagonism and agonism increased the number of striatal neural population spikes. The U‐shaped response is a pervasive phenomenon across neurobiology. For instance, both hyper‐ and hypo‐dopaminergic states drop cognitive performance in humans,^[^ ^39^ ^]^ and dopamine receptor activation can exert a dose‐dependent inhibitory or facilitatory effect on neuroplasticity.^[^ ^40^ ^]^ These studies suggest a logical explanation exists for the U‐shaped curve relationship between the mAChR activation and the striatal neural population activity found in this study.

Cholinergic signaling shapes the striatal neuronal activity mainly through the mAChRs. While all five subtypes of mAChRs are found in the striatum, M1 and M4 mAChRs are the most abundantly expressed subtypes in the striatal medium spiny neurons (MSNs). The inhibitory M4 mAChRs are more preferentially expressed in the direct pathway MSNs.^[^ ^41^ ^]^ Interestingly, the striatal M4 mAChRs are known to selectively enhance the excitability of the direct pathway MSNs through the calcium channel Ca_V_1.^[^ ^42^ ^]^ In contrast, the excitatory M1 mAChRs are predominantly expressed in the indirect pathway MSNs,^[^ ^43^ ^]^ and the striatal M1 mAChRs preferentially enhance both the somatic and dendritic excitability in the indirect pathway MSNs through the calcium channel Ca_V_2 and potassium channel Kir2.^[^ ^44^ ^]^ Therefore, the net effect of muscarinic receptor signaling is expected to enhance the excitability of the striatal neuronal population. However, it needs to be clarified how the relationship between the mAChR signaling and the striatal neural activity yielded a U‐shaped curve, warranting further studies to reconcile our findings and uncover the complex roles of muscarinic receptor signaling on the neuronal activity of the dorsal striatum.

### 3.4 Nicotine‐ or ChI‐Dependent Neural Plasticity in the Dorsal Striatum

Nicotine induces various neuroadaptive changes in the striatum, including at the neural population level. Our data revealed that nicotine increases the number of striatal neural population spikes, which are correlated with the number of neuronal cells firing in synchrony.^[^ ^20^ ^]^ The enhanced number of population spikes suggests that the probability of synchronous firing among the neuronal cells is higher, indicating that nicotine facilitates the synchronized activity of the dorsal striatum, as previously found.^[^ ^45^ ^]^

Additionally, our data show that the inhibition of striatal cholinergic interneurons enhanced the striatal neural activity. Supporting this finding, previous studies have shown that the striatal cholinergic interneurons can trigger a large GABA‐driven inhibitory response.^[^ ^1^, ^17^ ^]^ So, inhibiting striatal cholinergic interneurons is expected to enhance striatal neural activity. On the other hand, prior studies have also found that optogenetic inhibition of striatal ChIs decreases the excitability of MSNs.^[^ ^9^, ^46^ ^]^ Here, it is important to note that genetic inhibition leads to chronic silencing of neurons, while optogenetic inhibition leads to acute silencing. The difference in neuronal silencing strategy could vastly alter the response of neighboring neurons because chronic silencing leads to homeostatic plasticity.^[^ ^11b^ ^]^ In addition, distinctions exist between the mechanisms underlying neural population activity and single‐cell activity.^[^ ^20^ ^]^ These findings indicate that further studies are required to reconcile our electrophysiological findings with the previous studies.

Furthermore, it is unclear how in vivo nicotine priming led to an ex vivo nicotine‐induced increase in striatal neural activity and how the inhibition of striatal cholinergic interneurons blocks the nicotine's effect on striatal neural activity. At the very least, our data indicate that the striatal cholinergic interneurons are required for normal expression of the nicotine‐dependent neural plasticity in the dorsal striatum. Further studies are needed to identify the precise roles of striatal cholinergic interneurons in nicotine‐dependent striatal neural plasticity.

### 3.5 Striatal Acetylcholine‐Dopamine Balance in Nicotine Withdrawal

The acetylcholine‐dopamine balance hypothesis proposes that abnormal changes in the synchronized “pause” of the striatal cholinergic interneurons and the concurrent phasic firing of the nigrostriatal dopaminergic neurons underlie the striatal circuit dysfunctions in various brain disorders.^[^ ^43^ ^]^ The brief pause in striatal cholinergic interneurons and the phasic firing of nigrostriatal dopaminergic neurons temporally coincide with the learned motor response to reward‐related stimuli.^[^ ^47^ ^]^ This finding suggests that the striatal acetylcholine‐dopamine balance is an essential component of adaptive motor control.

Nicotine withdrawal substantially reduces the striatal dopamine release.^[^ ^48^ ^]^ Consequently, we found that inhibiting striatal cholinergic interneurons prevents the nicotine withdrawal‐induced decrease in striatal dopamine release. Also, the inhibition of striatal cholinergic interneurons reduced the evoked cholinergic tone in the dorsal striatum. These findings suggest that a reduction in the acetylcholine level can prevent the aberrant decrease in striatal dopamine release during nicotine withdrawal and implies that the striatal acetylcholine‐dopamine balance underlies the pathophysiology of nicotine withdrawal.

Our findings indicate that the nAChRs are at the interface of the acetylcholine‐dopamine balance and nicotine withdrawal. The striatal cholinergic interneurons can activate β2‐containing nAChRs expressed in the striatal dopaminergic terminals to cause local dopamine release.^[^ ^1^, ^22^ ^]^ During nicotine withdrawal, the signaling from β2‐containing nAChRs could be reduced through desensitization,^[^ ^37^ ^]^ which could decrease the basal level of striatal dopamine release. When the striatal cholinergic interneurons are inhibited, it could disrupt the nicotine‐dependent dopamine signaling in the dorsal striatum, thereby preventing the nicotine withdrawal‐related molecular changes, including the desensitization of the β2‐containing nAChRs. Further studies are warranted to validate this hypothesis and reveal the potential impact of acetylcholine‐dopamine balance on the pathophysiology of nicotine dependence.

### 3.6 miR‐137 as an Inhibitor of the Sodium Channel Subunit Na_V_1.1

In this study, we provided proof of concept that miRNAs can serve as an in vivo agent for the genetic inhibition of neurons, as previously shown.^[^ ^11^ ^]^ In the process, we revealed that the brain‐enriched miRNA miR‐137 inhibits the expression of sodium channel subunit Na_V_1.1. Previous studies have shown that miR‐137 is implicated in various brain diseases, including autism spectrum disorder,^[^ ^49^ ^]^ schizophrenia,^[^ ^50^ ^]^ intellectual disability,^[^ ^51^ ^]^ and drug withdrawal.^[^ ^52^ ^]^ Thus, further studies should explore the roles of miR‐137 in human brain diseases regarding the sodium channel subunit Na_V_1.1.

### 3.7 Neuropharmacological Properties of Procyclidine

Procyclidine has been shown to have an affinity for both the NMDA receptors (NMDARs) and nAChRs.^[^ ^53^ ^]^ Procyclidine mainly acts as a mAChR antagonist but also antagonizes NMDAR and nAChR. However, previous studies on mecamylamine indicate that the therapeutic effect of procyclidine on nicotine withdrawal is not due to NMDAR or nAChR antagonism. First, if the procyclidine‐dependent nAChR blockade were to take effect, as with mecamylamine, procyclidine would rather precipitate nicotine withdrawal signs or amplify the mecamylamine‐induced precipitation of nicotine withdrawal. Second, if the procyclidine‐dependent NMDAR blockade were therapeutic, mecamylamine should also ameliorate nicotine withdrawal signs because mecamylamine has a stronger potency for NMDAR antagonism than procyclidine.^[^ ^54^ ^]^ Therefore, altogether, these data indicate that procyclidine exerts its therapeutic effect on nicotine withdrawal through the inhibition of muscarinic receptor signaling.

### 3.8 Limitations

This study has several limitations. We used the ChAT(BAC)‐Cre mouse line to study nicotine withdrawal. A previous study has reported that the ChAT(BAC)‐Cre mouse line exhibits normal baseline behavior but shows deficits in nicotine self‐administration with higher expression levels of ChAT and vesicular acetylcholine transporter in the hippocampus.^[^ ^55^ ^]^ These findings suggest that the ChAT(BAC)‐Cre mouse line might not be adequate to study nicotine physiology, but our results contradict this claim. First, our behavioral data revealed that the ChAT(BAC)‐Cre mice normally express the somatic and affective signs of nicotine withdrawal and also exhibit nicotine reward and nicotine‐induced locomotor depression. These findings suggest that the ChAT(BAC)‐Cre mouse line is only inapt for studying a specific subset of nicotine‐dependent behaviors. Second, the experimental data on striatal electrophysiology, neurotransmitter release, and behavior obtained from the ChAT(BAC)‐Cre mice were readily replicated in the wild‐type C57BL/6J mice. These findings indicate that the molecular perturbations in the dorsal striatum of the ChAT(BAC)‐Cre mouse line do not deviate far from the wild‐type C57BL/6J mice. However, nicotine research with the ChAT(BAC)‐Cre mice should still be conducted with caution, as previously suggested. Characterizing nicotine‐dependent behaviors in the ChAT(BAC)‐Cre mice in conjunction with wild‐type mice would be essential for accurately interpreting the empirical data.

In addition, the gender effect was not considered in this study. The prevalence of tobacco smoking is approximately four times higher in males,^[^ ^56^ ^]^ but the physical burden of nicotine withdrawal is similar in both males and females.^[^ ^6^, ^57^ ^]^ Therefore, the pathophysiological significance of nicotine withdrawal syndrome in females must not be neglected. Future studies are warranted to investigate the gender similarities and differences in nicotine withdrawal^[^ ^58^ ^]^ concerning acute nicotine dependence, striatal cholinergic interneurons, and muscarinic receptor signaling.

Lastly, the striatal subregion‐specific effect has not been explored in this study. The dorsal striatum is largely divided into the dorsomedial and dorsolateral parts, which receive differential inputs from various cortices and are respectively involved in associative and sensorimotor processing.^[^ ^59^ ^]^ This tissue‐level distinction suggests that the dorsolateral striatum is presumably more involved in the ChI‐dependent control of motor dysfunctions, such as in Parkinson's disease in previous studies^[^ ^9^ ^]^ and nicotine withdrawal in this study. Importantly, a previous study demonstrated that the optogenetic activation of cholinergic interneurons in the dorsolateral striatum exacerbates L‐dopa‐induced dyskinesia.^[^ ^9d^ ^]^ This mounting evidence, in conjunction with the differential distribution of cholinergic interneurons across the dorsal striatum,^[^ ^60^ ^]^ implies that the functional distinction of cholinergic interneurons in the dorsolateral versus dorsomedial striatum merits further study in the future.

## 4 Conclusion

This study demonstrates that the striatal cholinergic interneurons gate the physical signs of nicotine withdrawal. The data on striatal neural activity and dopamine release suggest that muscarinic receptor signaling underlies the striatal cholinergic interneuron‐dependent control of physical nicotine withdrawal. Selective inhibition of the striatal cholinergic interneurons or muscarinic receptor signaling was proven as a potential therapeutic option against the physical signs of nicotine withdrawal. In summary, the data in this study provide important insights into acute nicotine dependence, nAChR‐mAChR interaction, acetylcholine‐dopamine balance, and the role of the dorsal striatum in nicotine withdrawal. Further research at circuit, synaptic, and molecular levels would further advance our understanding of the roles of striatal cholinergic interneurons in nicotine withdrawal.

## Conflict of Interest

The authors declare no conflict of interest.

## Author Contributions

B.K. performed project administration, conceptualization, methodology, funding acquisition, investigation, validation, visualization, formal analysis, data curation, wrote, reviewed edited the original draft. H.A.K. (HPLC‐ECD part) performed methodology, investigation, formal analysis. J.W. (patch‐clamp part) performed methodology, investigation, formal analysis, data curation. H.‐J.L. (LC‐MS/MS part) performed methodology, investigation, formal analysis, data curation. T.K.K. performed resources. H.M. and C.J.L. performed supervision. H.‐I.I. performed supervision, project administration, conceptualization, funding acquisition, resources, wrote, reviewed edited the original draft.

## Supporting information

Supporting Information

Supporting Information

## Data Availability

The data that support the findings of this study are available on request from the corresponding author. The data are not publicly available due to privacy or ethical restrictions.
