# Supplementary material for: Striatal Cholinergic Interneurons Control Physical Nicotine Withdrawal via Muscarinic Receptor Signaling
Source: Adv Sci (Weinh). 2024 Nov 3;11(47):2402274. doi: 10.1002/advs.202402274 (PMC11653618; doi:10.1002/advs.202402274)
Supplement: Supplementary file 1 — Supporting Information [file ADVS-11-2402274-s002.docx]

**Supplementary Material**

**
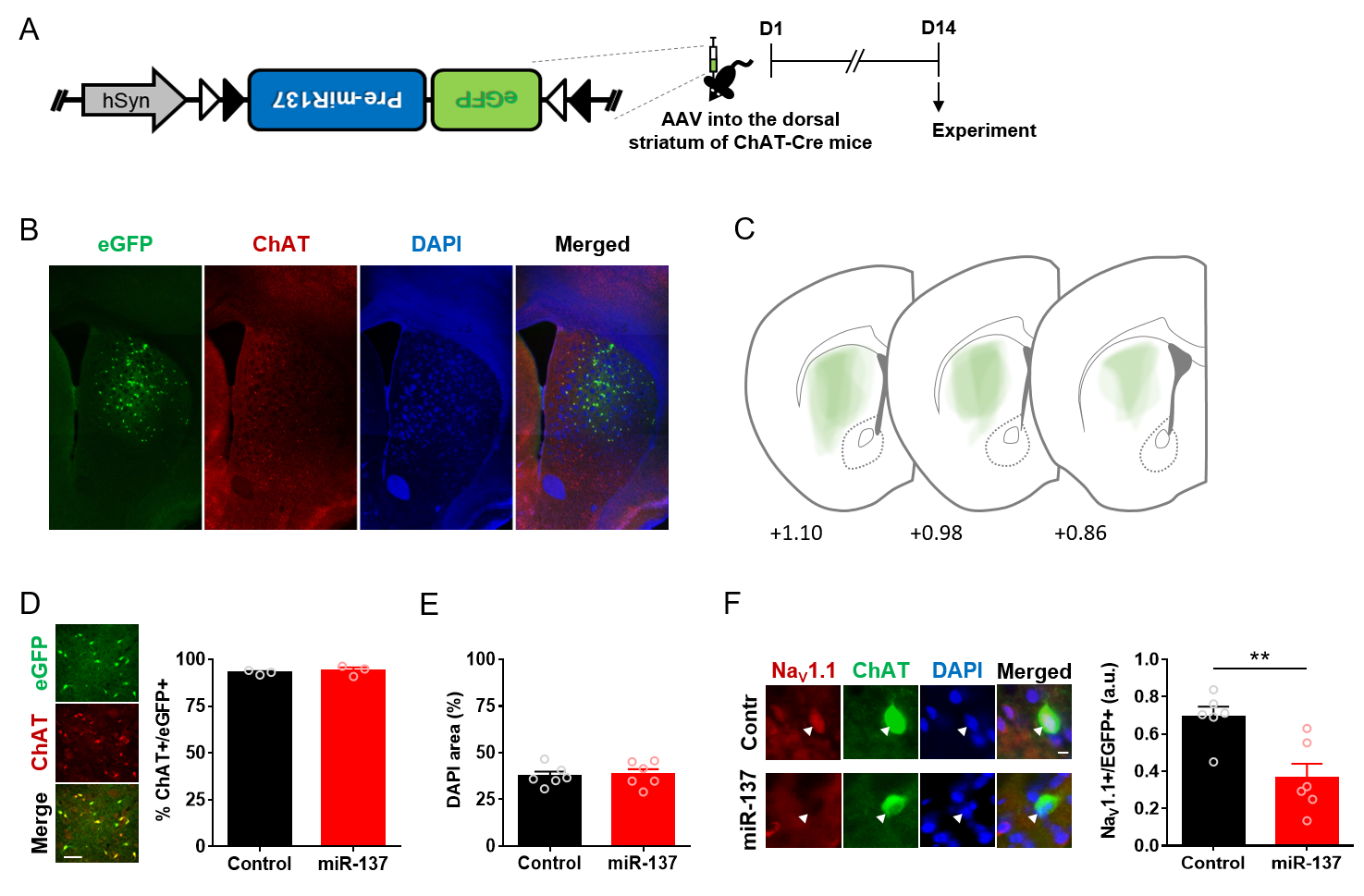
**

**Figure S1. Verification of AAV infectivity in the striatal cholinergic interneurons.**

**(A)** AAV construct for the Cre-dependent expression of miR-137 driven under the human synapsin promoter (hSyn), and simplified schedule for AAV injection into the dorsal striatum and histochemical analysis.

**(B)** A representative image of AAV spread in the whole dorsal striatum of ChAT-Cre mice.

**(C)** AAV spread (distribution of eGFP) in the dorsal striatum.

**(D)** AAV penetrance rate (n=3/group). Scale bar: 100 μm.

**(E)** DAPI area (n=5/group).

**(F)** (left) A representative image of immunohistochemistry showing reduced expression of Na_V_1.1 in the cholinergic neurons after miR-137 overexpression. Scale bar: 20 μm. (right) NaV1.1 expression level in the cholinergic interneurons after miR-137 overexpression (n=6/group) (Student’s *t*-test, ***p*=0.0056). a.u., arbitrary unit.

Error bars represent SEM.

**
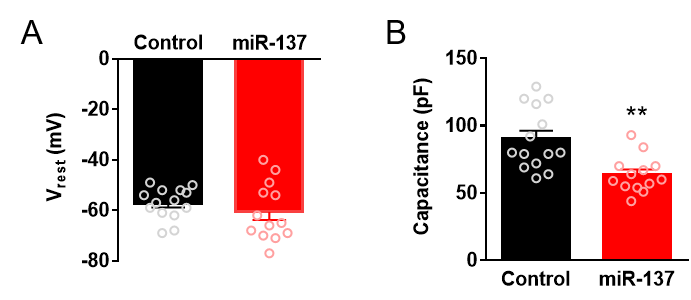
**

**Figure S2. ChI^GI^ reduces cell capacitance.**

**(A)** Impact of miR-137 on the resting membrane potential (V_rest_) (n=13~14/group).

**(B)** Impact of miR-137 on the cell capacitance (n=13~14/group) (Student’s *t*-test, ***p*=0.0013).

Error bars represent SEM.


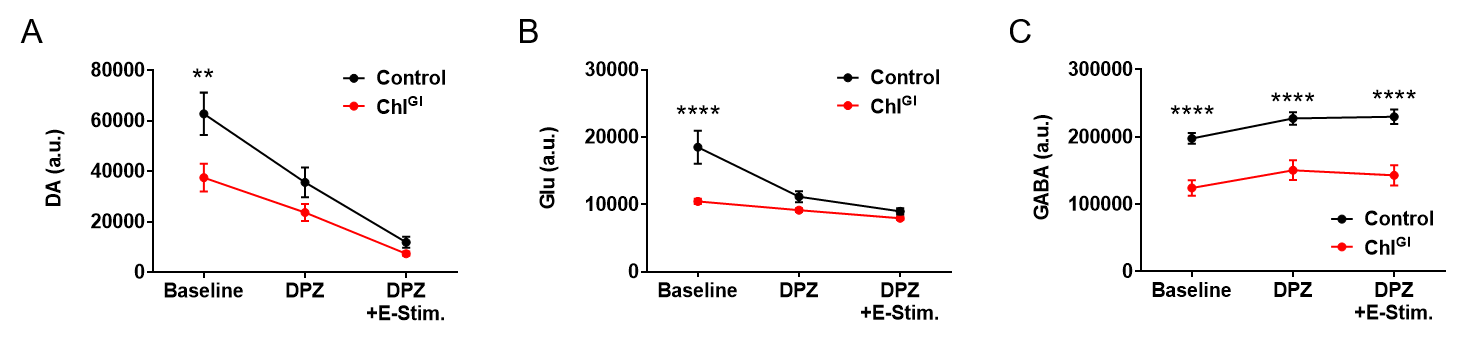


**Figure S3. ChI^GI^ reduces baseline release of dopamine, glutamate, and GABA.**

**(A)** Dopamine (DA) release from the striatal slices of Control and ChI^GI^ mice at baseline, after donepezil (DPZ) treatment, and after DPZ treatment with electrical stimulation (E-Stim.) (n=15~17/group) (Holm-Sidak’s *post-hoc* test, ***p*=0.0026). a.u., arbitrary unit.

**(B)** Glutamate (Glu) release from the striatal slices of Control and ChI^GI^ mice at baseline, after DPZ treatment, and after DPZ treatment with E-Stim. (n=15~17/group) (Holm-Sidak’s *post-hoc* test, *****p*<0.0001).

**(C)** GABA release from the striatal slices of Control and ChI^GI^ mice at baseline, after DPZ treatment, and after DPZ treatment with E-Stim. (n=15~17/group) (Holm-Sidak’s *post-hoc* test, *****p*<0.0001).

Error bars represent SEM.


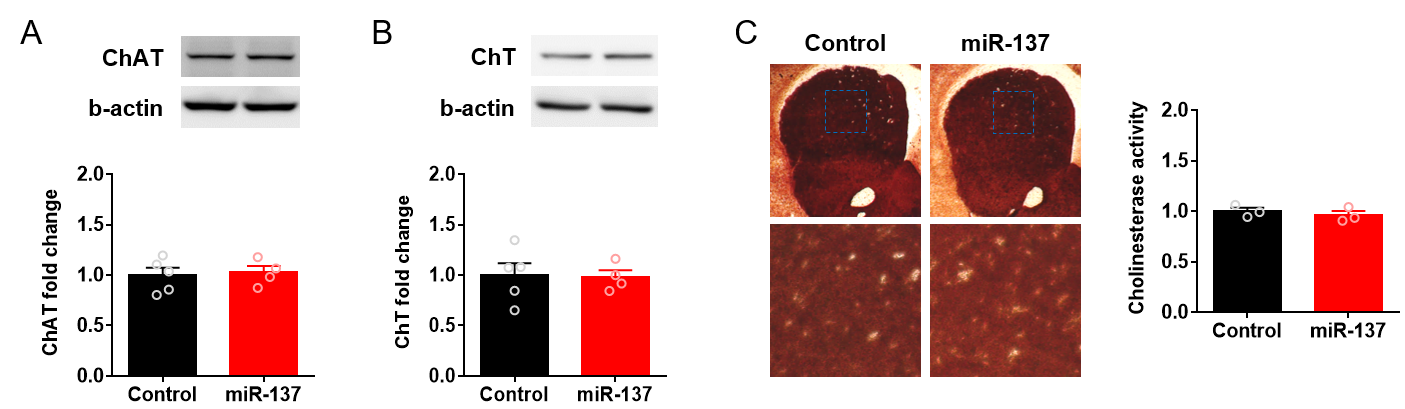


**Figure S4. ChI^GI^ exerts subtle effect on striatal acetylcholine synthesis machinery.**

**(A, B)** Immunoblotting of choline acetyltransferase (ChAT) and choline transporter (ChT) after miR-137 overexpression in the striatal ChIs (n=4~5/group).

**(C)** Cholinesterase assay after miR-137 overexpression in the striatal ChIs (n=3/group).

Error bars represent SEM.

**
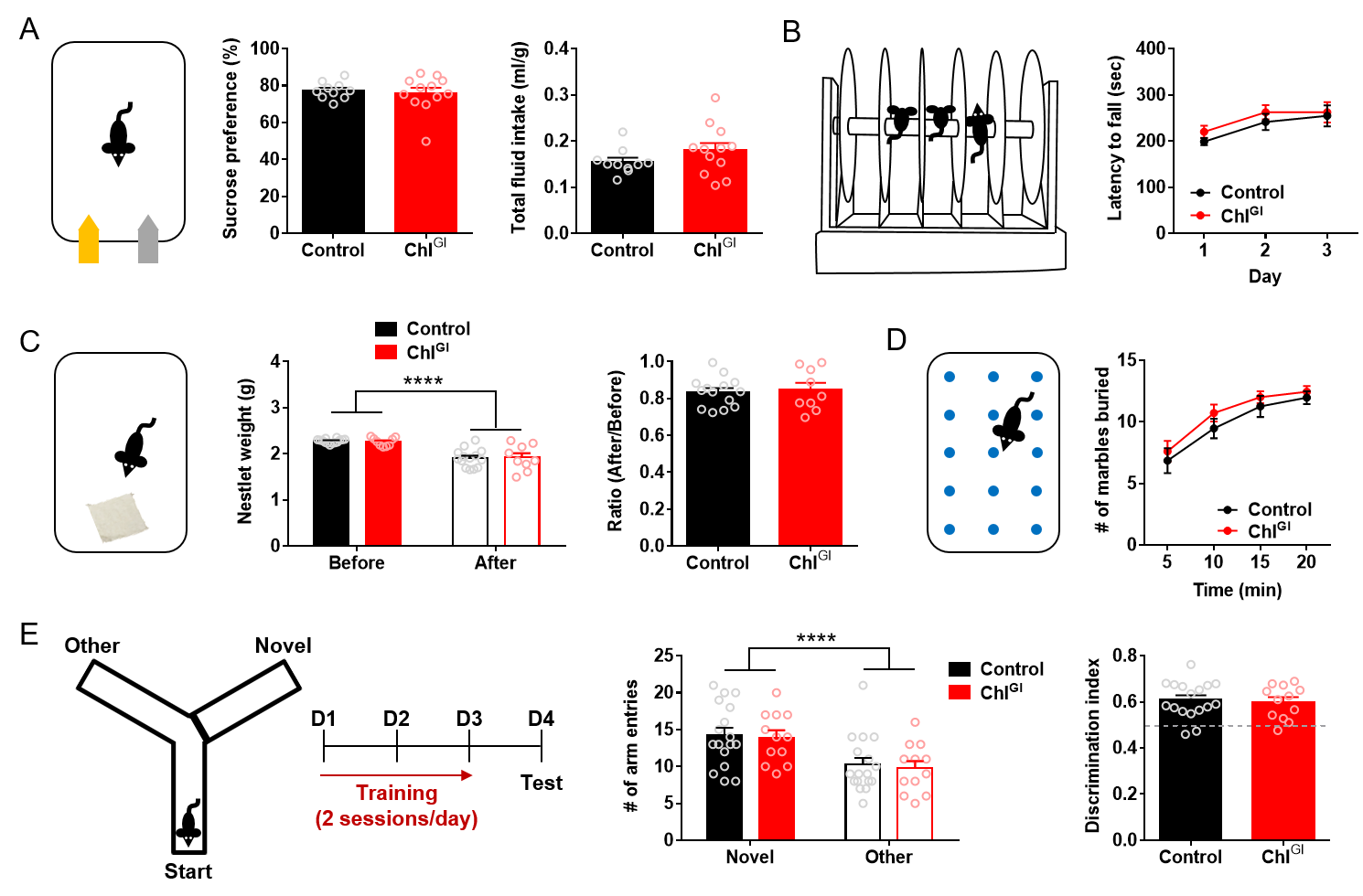
**

**Figure S5. ChI^GI^ exerts subtle effect on striatum-associated behaviors.**

**(A)** Sucrose preference test after genetic inhibition of striatal cholinergic interneurons (ChI^GI^) (n=10~12/group).

**(B)** Rotarod test after ChI^GI^ (n=8~9/group).

**(C)** Nestlet shredding test after ChI^GI^ (n=9~14/group) (Holm-Sidak’s *post-hoc* test, *****p*<0.0001).

**(D)** Marble burying test after ChI^GI^ (n=9~14/group).

**(E)** Y-maze spatial memory test after ChI^GI^ (n=12~17/group) (Holm-Sidak’s *post-hoc* test, *****p*<0.0001).

Error bars represent SEM.


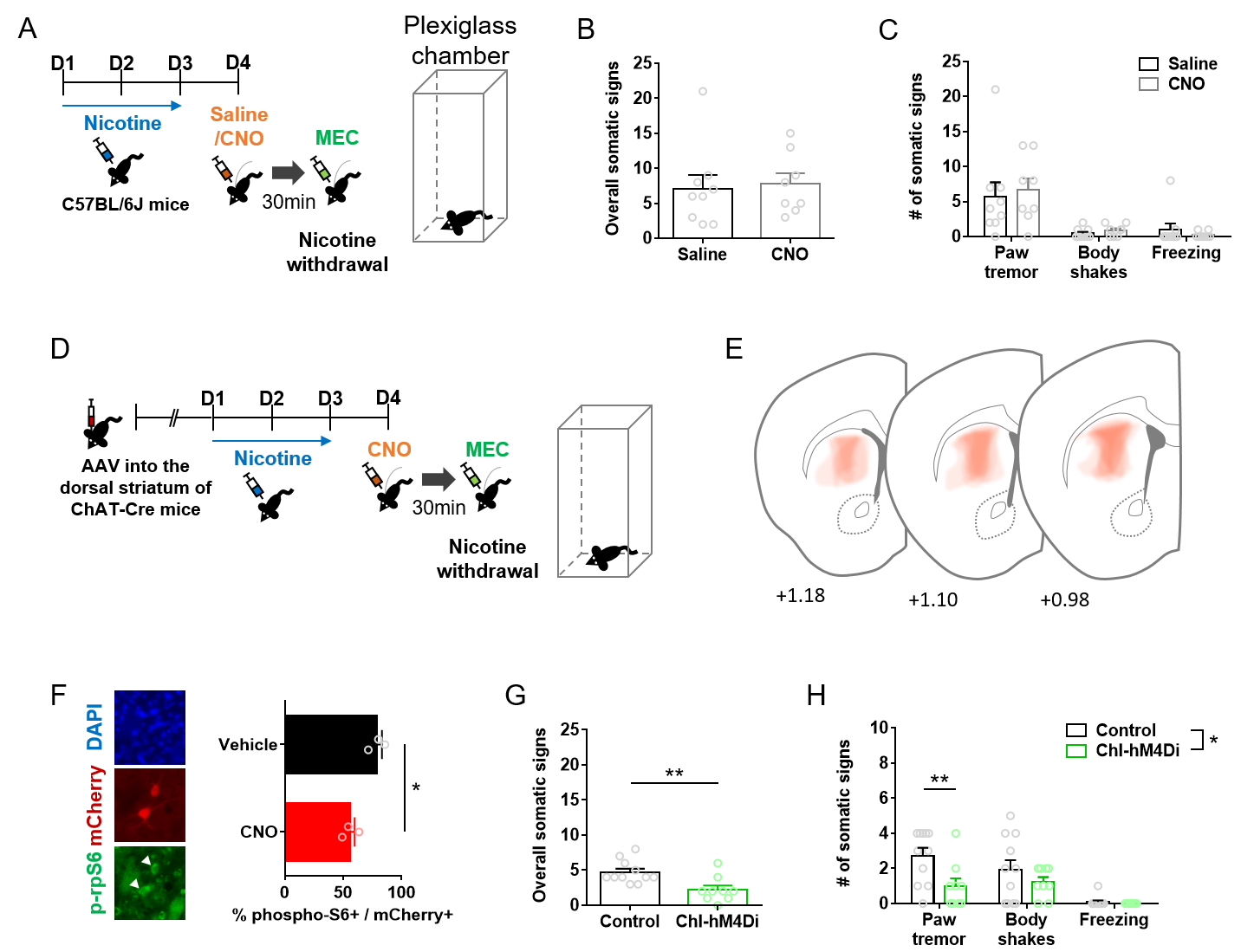


**Figure S6. Chemogenetic inhibition of striatal ChIs reduces the somatic signs of nicotine withdrawal.**

**(A)** Illustrated schedule for measuring the somatic signs of nicotine withdrawal after clozapine N-oxide (CNO) injection into wild-type C57BL/6 mice.

**(B)** Impact of CNO on the overall somatic signs of nicotine withdrawal (n=8~9/group).

**(C)** Impact of CNO on the individual somatic signs of nicotine withdrawal (n=8~9/group).

**(D)** Illustrated schedule for measuring the somatic signs of nicotine withdrawal after chemogenetic inhibition of striatal cholinergic interneurons. (below) representative image of mCherry signal showing hM4Di expression.

**(E)** A representative image of AAV spread in the dorsal striatum of ChAT-Cre mice.

**(F)** AAV spread (distribution of mCherry) in the dorsal striatum.

**(G)** Impact of chemogenetic inhibition of striatal cholinergic interneurons (ChI-hM4Di) on the overall somatic signs of nicotine withdrawal (n=9~11/group) (Student’s *t*-test, ***p*=0.0041).

**(H)** Impact of ChI-hM4Di on the individual somatic signs of nicotine withdrawal (n=9~11/group) (Holm-Sidak’s *post-hoc* test, ***p*=0.0068).

Error bars represent SEM.


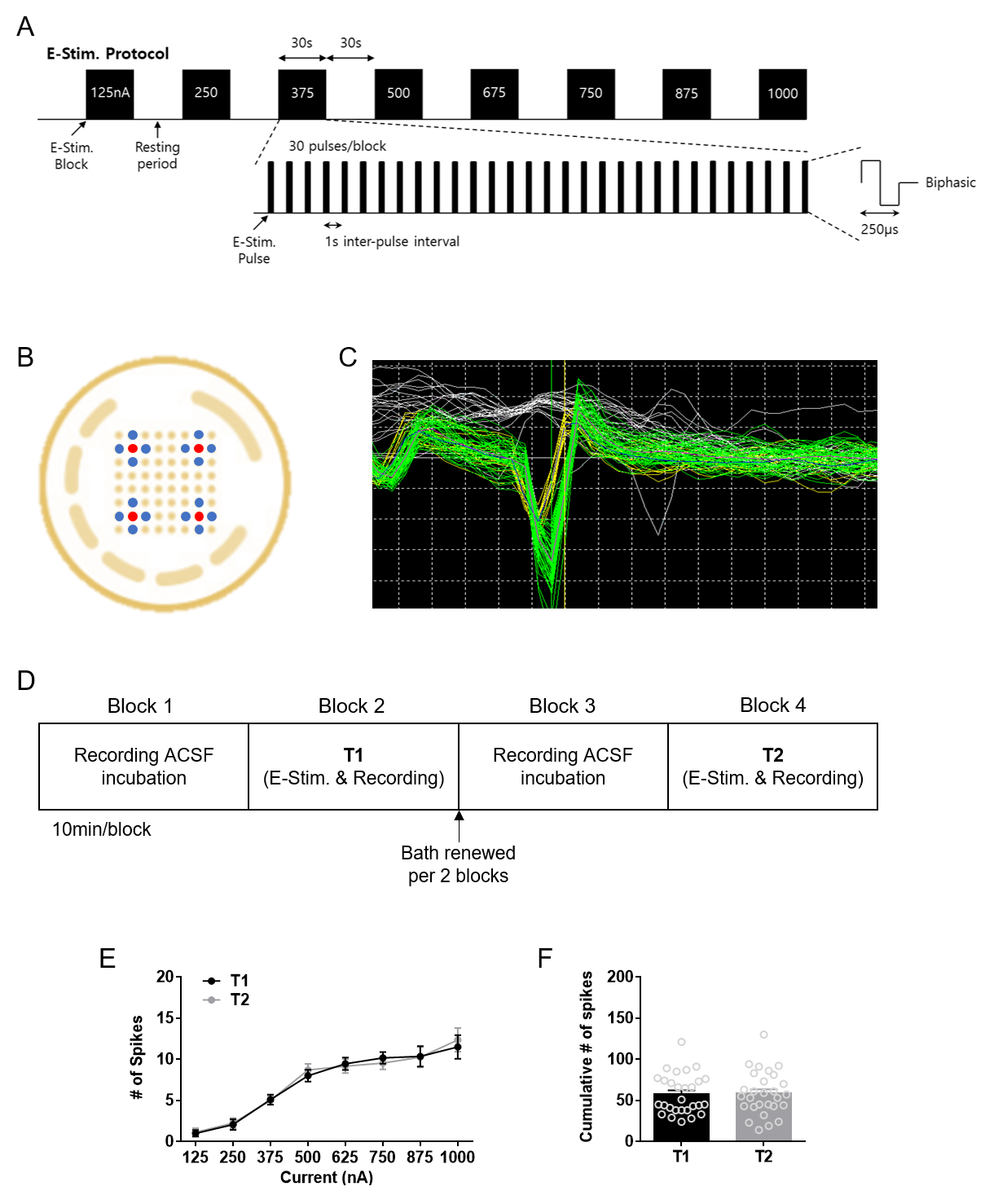


**Figure S7. Electrical stimulation-evoked neural population spike recording through multi-electrode array.**

**(A)** Electrical stimulation (E-Stim.) protocol in multi-electrode array (MEA) recording. The protocol consisted of 8 blocks, 30 s/block, with 30 s inter-block interval (resting period). A block consisted of 30 biphasic pulses with 1 s inter-pulse interval. 8 blocks were subjected to progressive increase in current intensity of the pulses in a block, from 125 to 1000 nA.

**(B)** E-Stim. protocol was applied onto 4 electrodes (red), and recording was conducted on 16 adjacent sites (blue). Only the stimulation electrodes and adjacent recording sites identified to be within the dorsal striatum were included for further analysis.

**(C)** A representative trace of striatal neural population spikes.

**(D)** MEA recording conducted sequentially at two time points (T1 and T2) in the dorsal striatum of wild-type C57BL/6 mice.

**(E)** The number of neural population spikes evoked in the dorsal striatum counted per block (30 pulses) (n=28).

**(F)** The cumulative number of neural population spikes in the dorsal striatum between the two time points (T1 and T2) (n=28).

Error bars represent SEM.


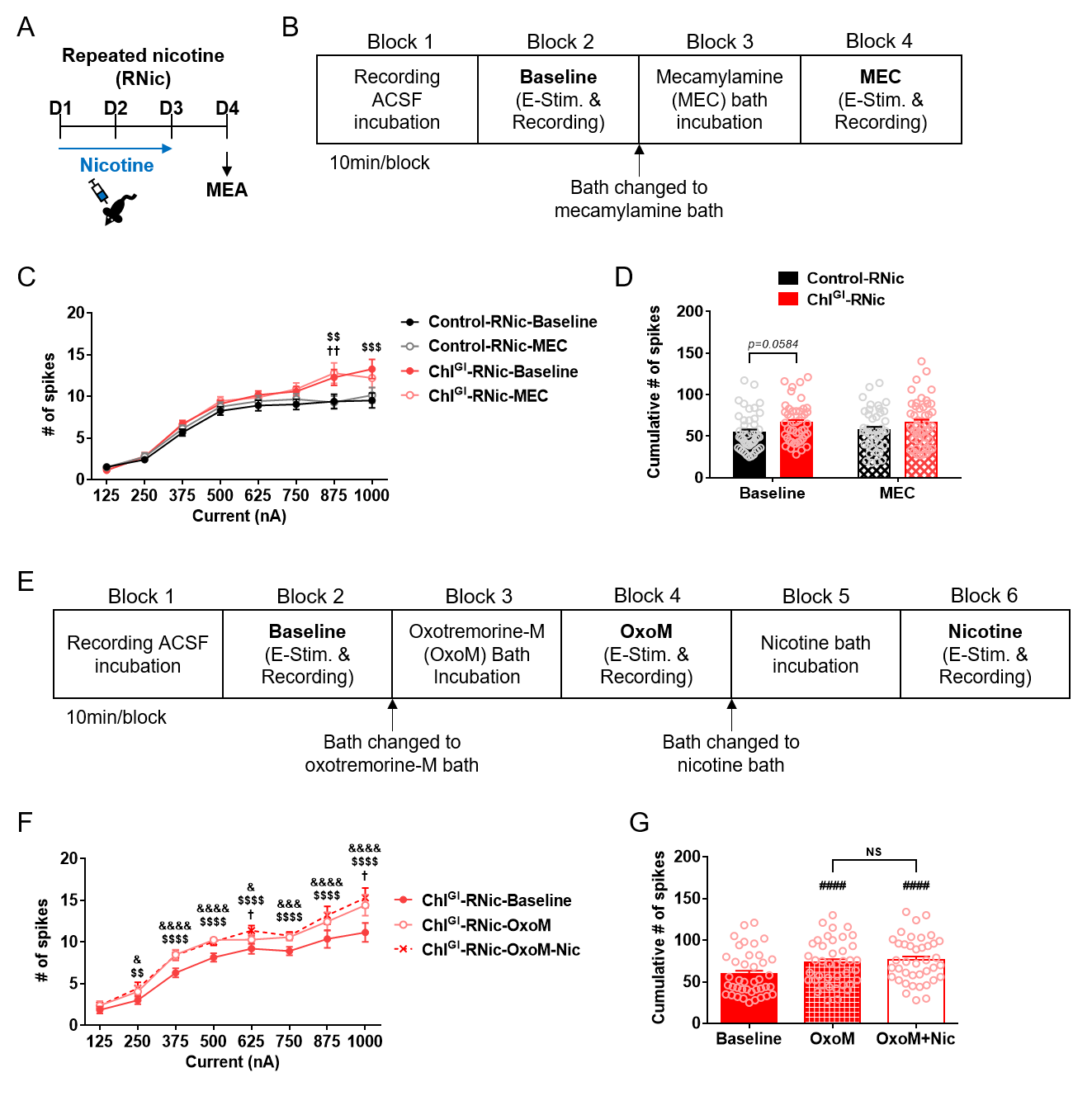


**Figure S8. Mecamylamine and oxotremorine-M differentially affect striatal population spikes after repeated nicotine exposure.**

**(A)** Illustrated schedule for repeated nicotine exposure (RNic) and MEA recording.

**(B)** MEA recording conducted before and after bath incubation of mecamylamine (MEC).

**(C)** Current-evoked neural population spikes in the dorsal striatum of Control and ChI^GI^ mice (n=44~47/group) (Holm-Sidak’s *post-hoc* test; compared groups and exact *p*-values are summarized in Table S1).

**(D)** Impact of mecamylamine on the neural population activity in the dorsal striatum of nicotine-experienced Control and ChI^GI^ mice (n=44~47/group).

**(E)** MEA recording conducted before and after bath incubation of oxotremorine-M (OxoM) and nicotine (Nic).

**(F)** Current-evoked neural population spikes in the dorsal striatum of ChI^GI^ mice (n=40/group) (Holm-Sidak’s *post-hoc* test; compared groups and exact *p*-values are summarized in Table S1).

**(G)** Impact of oxotremorine-M on the number of striatal neural population spikes (n=40/group) (Holm-Sidak’s *post-hoc* test, ^####^*p*<0.0001).

Error bars represent SEM. Statistical significance for **(C)** and **(F)** are summarized in Table S1.


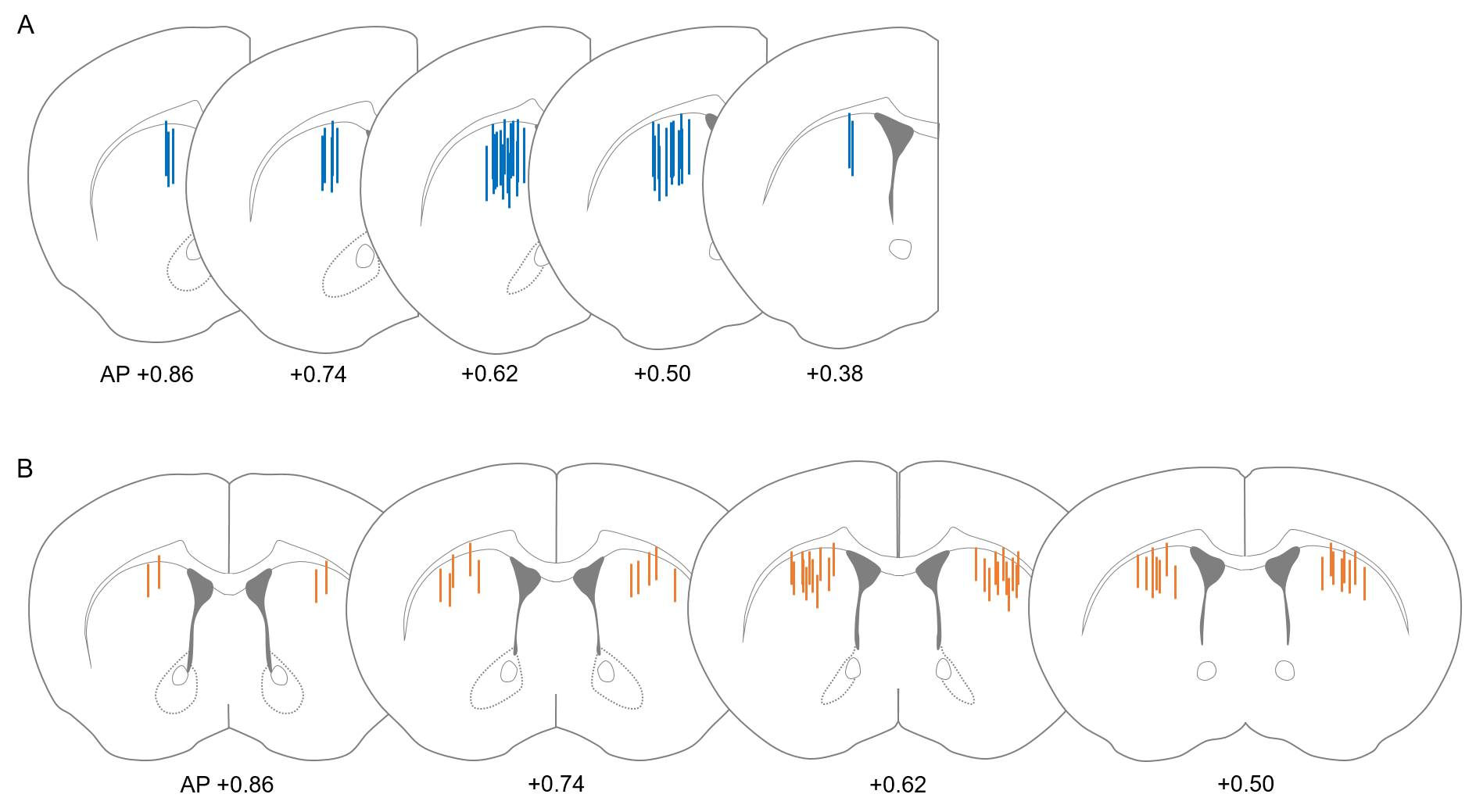


**Figure S9. Microdialysis probe and cannula implantation site verification.**

**(A)** Blue lines indicate the sites of microdialysis probe implantation.

**(B)** Orange lines indicate the sites of cannula implantation.

| **Figure panel** | **Comparison** | **Current (nA)** | **Indication** | ***p*-value** |
| --- | --- | --- | --- | --- |
| Fig. 3C | Control-Naïve-Baseline vs. Control-Naïve-Nicotine | 500 | && | 0.0089 |
|  |  | 1000 | && | 0.0057 |
|  | ChI^GI^-Naïve-Baseline vs. ChI^GI^-Naïve-Nicotine | 375 | αα | 0.0013 |
|  |  | 500 | ααα | 0.0001 |
|  |  | 625 | ααα | 0.0001 |
|  |  | 750 | ααα | 0.0006 |
|  |  | 1000 | αααα | < 0.0001 |
|  | Control-Naïve-Baseline vs. ChI^GI^-Naïve-Baseline | 875 | $$ | 0.0046 |
|  | Control-Naïve-Nicotine vs. ChI^GI^-Naïve-Nicotine | 625 | † | 0.0264 |
|  |  | 750 | † | 0.0101 |
|  |  | 875 | †† | 0.001 |
|  |  | 1000 | ††† | 0.0004 |
| Fig. 3E | Control-RNic-Baseline vs. Control-RNic-Nicotine | 375 | & | 0.0157 |
|  |  | 500 | & | 0.0157 |
|  |  | 625 | &&&& | < 0.0001 |
|  |  | 875 | &&&& | < 0.0001 |
|  |  | 1000 | &&&& | < 0.0001 |
|  | Control-RNic-Baseline vs. ChI^GI^-RNic-Baseline | 625 | $ | 0.0402 |
| Fig. 4C | Naïve-Baseline vs. Naïve-Proc | 375 | &&&& | < 0.0001 |
|  |  | 500 | && | 0.0026 |
|  |  | 625 | &&& | 0.0009 |
|  |  | 750 | &&& | 0.0009 |
|  |  | 875 | &&&& | < 0.0001 |
|  |  | 1000 | &&&& | < 0.0001 |
|  | Naïve-Baseline vs. Naïve-Proc-Nic | 375 | $$$$ | < 0.0001 |
|  |  | 500 | $$$$ | < 0.0001 |
|  |  | 625 | $$$ | 0.0003 |
|  |  | 750 | $$$ | 0.0002 |
|  |  | 875 | $$$$ | < 0.0001 |
|  |  | 1000 | $$$$ | < 0.0001 |
|  | Naïve-Proc vs. Naïve-Proc-Nic | 500 | †† | 0.0026 |
|  |  | 875 | † | 0.0408 |
|  |  | 1000 | †† | 0.0019 |
| Fig. 4E | RNic-Baseline vs. RNic-Proc | 250 | && | 0.0015 |
|  |  | 375 | & | 0.0201 |
|  |  | 500 | & | 0.0353 |
|  |  | 625 | &&& | 0.001 |
|  |  | 750 | && | 0.0019 |
|  |  | 1000 | & | 0.0413 |
|  | RNic-Baseline vs. RNic-Proc-Nic | 250 | $$$ | 0.0005 |
|  |  | 375 | $$$ | 0.0003 |
|  |  | 500 | $ | 0.0438 |
|  |  | 625 | $$ | 0.005 |
|  |  | 750 | $$ | 0.0072 |
| Fig. S8C | Control-RNic-Baseline vs. ChI^GI^-RNic-Baseline | 875 | $$ | 0.0099 |
|  |  | 1000 | $$$ | 0.0003 |
|  | Control-RNic-MEC vs. ChI^GI^-RNic-MEC | 875 | †† | 0.0034 |
| Fig. S8F | ChI^GI^-RNic-Baseline vs. ChI^GI^-RNic-OxoM | 250 | & | 0.0264 |
|  |  | 375 | &&&& | < 0.0001 |
|  |  | 500 | &&&& | < 0.0001 |
|  |  | 625 | & | 0.0157 |
|  |  | 750 | &&& | 0.0001 |
|  |  | 875 | &&&& | < 0.0001 |
|  |  | 1000 | &&&& | < 0.0001 |
|  | ChI^GI^-RNic-Baseline vs. ChI^GI^-RNic-OxoM-Nic | 250 | $$ | 0.0018 |
|  |  | 375 | $$$$ | < 0.0001 |
|  |  | 500 | $$$$ | < 0.0001 |
|  |  | 625 | $$$$ | < 0.0001 |
|  |  | 750 | $$$$ | < 0.0001 |
|  |  | 875 | $$$$ | < 0.0001 |
|  |  | 1000 | $$$$ | < 0.0001 |
|  | ChI^GI^-RNic-OxoM vs. ChI^GI^-RNic-OxoM-Nic | 625 | † | 0.0157 |
|  |  | 1000 | † | 0.0344 |

**Table S1. Summary of statistical significance for the MEA data per step current.**

*p*-values were derived from one-way or two-way RM ANOVA followed by Holm-Sidak’s *post-hoc* test.

**Materials and Methods**

*1. Animals*

B6.FVB(Cg)-Tg(Chat-cre)GM60Gsat/Mmucd (ChAT-Cre or ChAT-BAC-Cre) transgenic mice were originally obtained from the MMRRC (<https://www.mmrrc.org/catalog/sds.php?mmrrc_id=30869>) and bred in wild-type x hemizygous transgenic mating pairs. The ChAT-Cre mouse line had been backcrossed onto the wild-type C57BL/6J background for more than 20 generations. B6.Cg-Tg(Camk2a-cre)T29-1Stl/J (Camk2a-Cre) transgenic mice were originally obtained from the Jackson Laboratory (#005359). The Camk2a-Cre mouse line had been backcrossed onto the wild-type C57BL/6J background for more than 10 generations. C57BL/6J mice were purchased (Daehan Bio Link, Daejeon, Republic of Korea; KIST SPF animal facility, Seoul, Republic of Korea) a week before experimentation.

8~16-weeks-old male ChAT-Cre mice, 8-weeks-old male Camk2a-Cre mice, and 8-weeks-old male C57BL/6J mice were used for experimentation. All mice were housed in plastic cages with metal wire grids and were maintained under a 12-hour reversed light/dark cycle (lights off at 7:00 AM). Mice had *ad libitum* access to food and drinking water.

Only prior to the sucrose preference test, mice had limited access to drinking water (see “Sucrose preference test” section in Materials and Methods). Mice were housed in groups of 2~4, but were single-housed after the intrastriatal implantation of cannulas (see “Cannula implantation” sections in Materials and Methods).

For ethical considerations regarding animal experimentation, all procedures regarding the handling and use of animals in this study were conducted as approved by the Institutional Animal Care and Use Committee (IACUC) of Korea Institute of Science and Technology (KIST).

*2. Stereotaxic surgery*

For the stereotaxic surgery, mice were anesthetized with an intraperitoneal (i.p.) injection of ketamine/xylazine mixture (120 and 8 mg/kg, respectively) and mounted on a stereotaxic apparatus (Kopf Instruments, CA, USA). A single booster injection (ketamine, 120 mg/kg) was made during microdialysis probe or cannula implantation surgery.

*2.1. Intrastriatal injection of AAV*

For intrastriatal injection of AAV, AAV-DJ-hSyn-DIO-mir137-eGFP or AAV-hSyn-DIO-hM4Di-mCherry was cloned and packaged (~1.0 x 10^14^ GC/ml) (KIST virus facility, Seoul, Republic of Korea). For control, AAV-DJ-hSyn-DIO-eGFP or AAV-hSyn-DIO-mCherry was used. Microinjections were conducted with a 10-μL glass syringe (#80383; Hamilton, NV, USA) and a programmable syringe pump (NE-4000; New Era Pump Systems, NY, USA). Microinjections were made into both hemispheres of the whole dorsal striatum at the following coordinates: anteroposterior (AP) +1.1 mm; mediolateral (ML) ±1.65 mm; dorsoventral (DV) −3.1, −2.7 and −2.3 mm below dura. Three microinjections were delivered into each hemisphere of the whole dorsal striatum, with the injection volumes of 0.2, 0.7, and 0.7 μl per coordinate. The injection rate was 0.1 μl/min, and the microneedle was held in position for 2 min after each injection. Animals were used for experimentation at least 2 weeks after intrastriatal AAV injection.

*2.2. Cannula implantation*

Two stainless steel cranial nails were implanted at sites away from the cannula implantation site(s), without impacting the cortex beneath. The cranial nails were used to anchor the dental cement and cannula.

For the microdialysis experiment, an intracerebral guide cannula microdialysis probe with 1-mm membrane and 5-mm cannula (MD-2255; BASi, IN, USA) was unilaterally implanted into the dorsal striatum at the following coordinates: anteroposterior (AP) +0.7 mm; mediolateral (ML) ±1.65 mm; dorsoventral (DV) −2.1 mm below dura. The left and right hemispheres were counterbalanced for microdialysis probe implantation.

For the intrastriatal drug administration, a 22-gauge 6-mm guide cannula (C313G-5UP; PlasticsOne, VA, USA) was bilaterally implanted into the dorsal striatum at the following coordinates: anteroposterior (AP) +0.7 mm; mediolateral (ML) ±1.65 mm; dorsoventral (DV) −2.1 mm below dura. The “fits-on” cannula dummy was made with an end-sealed 7-mm internal cannula.

Cannulae were secured in position using dental cement from the Ortho-Jet^TM^ Package (1334CLR; Lang, IL, USA). After surgery, mice were single-housed and examined once a day for a duration of 3 days to monitor the overall health status. Experimentation was conducted after at least 1 week of recovery from cannula implantation. Cannula implantation sites were verified after experimentation, and only the animals with accurate cannula placement were used for data analysis.

*3. Bioinformatics*

The bioinformatics pipeline was implemented for the prediction of miRNAs targeting voltage-gated sodium channels (VGSCs), specifically the alpha subunits Na_V_1.1 (SCN1A) and Na_V_1.6 (SCN8A) which are known to be highly expressed in the striatal ChIs. Through miRNA target prediction using TargetScanHuman v.8.0 (using only the conserved sites for miRNA families broadly conserved among vertebrates) [1] and MicroT-CDS v5.0 (miTG score > 0.85) [2], two miRNAs were predicted to target SCN1A and SCN8A. Then, we selected the brain-enriched and evolutionarily conserved miRNA, miR-137, as the agent for the genetic inhibition of striatal ChIs.

*4. Luciferase assay*

miRNA 3´UTR target expression clone for Human SCN1A (NM_001165963.1) (HmiT056442-MT05, GeneCopoeia, MD, USA), miRNA 3´UTR target expression clone for Human SCN8A (NM_014191.2) (HmiT016604-MT05, GeneCopoeia), human pre-microRNA expression construct Lenti-miR-137 (PMIRH137PA-1, System Biosciences), and appropriate control vectors were used for the luciferase assay. Either the SCN1A-3’UTR or SCN8A-3’UTR vector was transfected together with Lenti-miR-137 vector or a scrambled control vector to the HEK293TN cell line in a 12-well cell culture plate using the EndoFectin Lenti transfection reagent (GeneCopoeia). Media was changed at 16 hours after transfection and were collected at 48 hours after transfection for analysis.

Luciferase assay was conducted using Secrete-Pair^TM^ Dual Luminescence Assay Kit (LF031; GeneCopoeia) according to the manufacturer’s protocol. The activities of *Gaussia* luciferase and secreted alkaline phosphatase were gauged as a measure of absorbance reading with the Synergy HTX Multi-Mode Microplate Reader (BioTek, VT, USA). Then the luciferase activity was normalized to the alkaline phosphatase activity.

*5. Immunohistochemistry*

Mice were anesthetized with Avertin (2,2,2-tribromoethanol, 250 mg/kg; Sigma-Aldrich). For Na_V_1.1 immunohistochemistry, transcardial perfusion was performed with 60 ml of 1X PBS followed by 30 ml of 2% paraformaldehyde in 1X PBS, and the whole brain was immediately isolated and post-fixed in 2% paraformaldehyde in 1X PBS for 1 hour at 4 °C. For other immunohistochemical targets, transcardial perfusion was performed with 60 ml of 1X PBS followed by 30 ml of 4% paraformaldehyde in 1X PBS, and the whole brain was immediately isolated and post-fixed in 4% paraformaldehyde in 1X PBS for overnight at 4 °C. The brain was briefly washed in 1X PBS and dehydrated in 30% sucrose in 1X PBS at 4 °C with gentle agitation until submersion. Then, the brain was embedded in OCT compound and immediately frozen in -80 °C freezer until cryosectioning.

The brain was coronally cryosectioned to a 30-μm thickness in a -20 °C cryostat. Free-floating sections were gently rinsed in 1X PBS, followed by incubation in ice-cold 1X PBS at 4 °C for 5 min. For Na_V_1.1 immunohistochemistry, sections were incubated in a pepsin solution (R2283; Sigma-Aldrich, MO, USA) at 37 °C for 20 seconds, and were quickly, but gently washed with ice-cold 1X PBS. Washed sections were blocked in 1X PBS-T (0.1% Tween-20) containing 10% normal goat serum (NGS) for 2 hours at room temperature, then were incubated for 48 hours at 4 °C with the appropriate combinations of primary antibodies: Rabbit anti-ChAT (1:2000) (ab178850; Abcam, UK), mouse anti-Na_V_1.1 (1:100) (75-023; NeuroMab, UC Davis, CA, USA), chicken anti-mCherry (1:500) (ab205402; Abcam), or rabbit anti-phospho-S6 ribosomal protein (Ser235/236) (#2211; Cell Signaling Technology, MA, USA). Subsequently, sections were washed and incubated for 2 hours at room temperature with the appropriate combinations of secondary antibodies: Goat anti-rabbit IgG Alexa Fluor 594 (1:400) (Thermo Fisher Scientific, MA, USA), goat anti-rabbit IgG Alexa Fluor 488 (1:400), goat anti-mouse IgG Alexa Fluor 594 (1:400), or goat anti-chicken IgG Alexa Fluor 594 (1:400). Finally, sections were washed and gently mounted on VECTASHIELD HardSet Antifade Mounting Medium with DAPI (Vector Laboratories, CA, USA). Images were shot with FluoView FV1000 (Olympus, Tokyo, Japan) or EVOS M7000 Imaging System (Thermo Fisher Scientific), and visualized using Zen software (Carl Zeiss, Oberkochen, Germany). DAPI area and cell fluorescence (Na_V_1.1 signal or phospho-S6 ribosomal protein signal) were analyzed using ImageJ software (NIH, USA).

*6. Patch-clamp recording*

Animals were anesthetized with isoflurane and isolated brains were submerged in ice-cold ACSF solution (130mM NaCl, 24mM NaHCO_3_, 1.25mM NaH_2_PO_4_, 3.5mM KCl, 1.5mM CaCl_2_, 1.5mM MgCl_2_, and 10mM D(+)-glucose, pH 7.4). 300-μm slices were cut using a vibratome (DSK Linear Slicer, Kyoto, Japan) oxygenated in ACSF at room temperature for 1 hour, and then acclimated at room temperature with continuous perfusion with ASCF solution (2ml/min). Slices were placed in the recording chamber and target cells (eGFP+) were identified via an upright Olympus microscope with a 60X water immersion objective with infrared differential interference contrast optics. Whole-cell recording was performed with pCLAMP10 and MultiClamp 700B amplifier (Axon Instrument, Molecular Devices) at room temperature from cholinergic interneurons. The holding potential was -60 mV. Pipette resistance was in the range of 5-8 MOhm. The pipette was filled with an internal solution (in mM): 140 K-gluconate, 10 HEPES, 7 NaCl, and 2 MgATP adjusted to pH 7.4 with CsOH for action potential measurements. All holding potential values stated are after correction for the calculated junction potential offset of 14 mV. Electrical signals were digitized and sampled at 50 μs intervals with the Digidata 1550B and Multiclamp 700B amplifier (Molecular Devices, CA, USA) using pCLAMP 10.7 software. Data were filtered at 2 kHz. The recorded current was analyzed with the Minianalysis (Synaptosoft) software. Off-line analysis was carried out using Clampfit, Minianalysis, and Excel software.

*7. Multi-electrode array (MEA)*

Mice were anesthetized with Avertin, and were transcardially perfused with 30 ml of ice-cold oxygenated NMDG-ACSF composed of (in mM) 92 NMDG, 25 glucose, 5 Na L-ascorbate, 2.5 KCl, 1.25 NaH_2_PO_4_, 2 thiourea, 10 MgCl_2_, 30 NaHCO_3_, 20 HEPES, 3 Na pyruvate, and 0.5 CaCl_2_ at pH 7.4. Coronal brain slices (300 mm) containing the dorsal striatum were acutely prepared using a vibratome (VT1000S; Leica Biosystems, Wetzlar, Germany) in cold oxygenated NMDG-ACSF. Subsequently, the isolated brain slices were bisected and moved to a chamber filled with oxygenated NMDG-ACSF at 32 °C for 30 min, followed by room temperature (25 °C) oxygenated standard ACSF composed of (in mM) 92 NaCl, 25 glucose, 5 Na L-ascorbate, 2.5 KCl, 1.25 NaH_2_PO_4_, 2 thiourea, 2 MgCl_2_, 30 NaHCO_3_, 20 HEPES, 3 Na pyruvate, and 2 CaCl_2_ at pH 7.4 at room temperature for 60 min prior to MEA recording.

For MEA recording with acute brain slice, the “Acute Brain Slice Protocol” from Axion Biosystems was used with minor modifications [3]. The brain slices were gently moved onto the 0.1% polyethylenimine-coated wells of CytoView MEA 6 plate (M384-tMEA-6W WHITE; Axion Biosystems) using fire-polished pasteur pipette. The brain slice was positioned onto the electrodes using small brush and the position was verified using light microscope. Then the media was suctioned from wells, a pre-wet Harp Slice Grids (HSG-MEA-5CD; ALA Scientific, Farmingdale, NY, USA) was gently placed onto the brain slice using forceps, and the wells were gently replenished with 100 μl of recording ACSF composed of (in mM) 124 NaCl, 12.5 glucose, 2.5 KCl, 1.25 NaH_2_PO_4_, 2 MgCl_2_, 24 NaHCO_3_, 20 HEPES, and 2 CaCl_2_ at pH 7.4. From media suctioning to replenishing took <2 min for each well. Then, the media was suctioned from wells and the wells were replenished again with 100 μl of recording ACSF. Then the CytoView MEA 6 plate was moved into Maestro Edge MEA System (Axion Biosystems, GA, USA) for recording.

Recording chamber in Maestro Edge was set at 32 °C and continuously aerated with carbogen. MEA data acquisition and electrical stimulation were accomplished through AxIS Navigator (Axion Biosystems). An electrical stimulation session consisted of 8 blocks with 30-s inter-block interval, 30 pulses/block with 1-s inter-pulse interval, with each pulse consisting of a biphasic waveform lasting 250 μs. Progressive increment in the stimulation intensity was applied throughout the blocks, from 125 to 1000 nA, to evoke neural population spikes which corresponds to the neuronal cells firing in synchrony [4]. Briefly, the neural population spikes are generated from the summation of electrical currents during synchronous firing of action potentials. The neural population spikes reflect both the macroscopic (volume propagation of currents in the extracellular space) and subcellular (localized spread and magnitude of axial and transmembrane currents) factors in field potentials [4a]. It has been found that the shape and timing of neural population spikes are correlated with the unit spikes (single-neuron activity), and that the size of neural population spikes are correlated with the stimulus strength (e.g. electrical stimulation) [4b].

Acquired data were post-processed through AxionDataExportTool (Axion Biosystems) and Offline Sorter V4 (Plexon, TX, USA). The number of evoked population spikes were counted in each of the four recording sites immediately near the stimulation electrode. Recording sites with stimulation-response fidelity <10% (spikes evoked in less than 10% of all stimulation events) in any electrical stimulation session were excluded from analysis.

(-)-Nicotine ditartrate (#3546; Tocris Bioscience, UK) was dissolved in distilled water to 200 μM stock (1000x) and stored in -80 °C. Nicotine stock was diluted in recording ACSF to 200 nM just prior to MEA recording. Procyclidine hydrochloride (P3794; Sigma-Aldrich) was dissolved in distilled water to a 2 mM stock (20x) and stored in -20 °C. Procyclidine stock was diluted in recording ACSF to 100 μM just prior to MEA recording. Mecamylamine hydrochloride (M9020; Sigma-Aldrich) was dissolved in distilled water to 2 mM stock (200x) and stored in -20 °C. Mecamylamine stock was diluted in recording ACSF to 10 μM just prior to MEA recording. Oxotremorine-M (#1067; Tocris Bioscience) was dissolved in distilled water to a 10 mM stock (1000x) and stored in -20 °C. Oxotremorine-M stock was diluted in recording ACSF to 10 μM just prior to MEA recording.

*8. MEA ACSF bath for neurotransmitter analysis*

Brain slice preparation to MEA recording was conducted as previously described. Donepezil hydrochloride (#4385; Tocris Bioscience) was dissolved in distilled water to 1 mM stock (100x) and stored in -80 °C. Donepezil stock was diluted in recording ACSF to 10 μM just prior to bath change. Fixed stimulation intensity (at 500 nA) was applied throughout the blocks to stimulate the dorsal striatum. The collected ACSF samples were subjected to LC-MS/MS for neurotransmitter analysis.

Acetylcholine, dopamine, glutamate, and GABA were obtained from Sigma-Aldrich. LC-MS-grade water and acetonitrile were supplied from Fisher Scientific (Pittsburgh, PA, USA), and formic acid was sourced from Wako Chemical (Tokyo, Japan). To create the calibration curve, working solutions equivalent to 10, 50, 100, 1,000, 10,000, 10,000, 100,000, and 500,000 fg/µL were prepared using a pooled stock solution of acetylcholine, dopamine, glutamate, and GABA. Calibration standards and ACSF samples were prepared by adding 4 µL of the appropriate working solution, and ACSF to 16 µL of distilled water. These mixtures were then vortexed and centrifuged at 16,000 g for 15 min at 4 ℃. After centrifugation, 18 µL of the supernatant was transferred to vials for LC-MS/MS analysis.

The absolute quantification of acetylcholine, dopamine, glutamate, and GABA was performed with the Altis plus mass system equipped with Vanquish (Thermo Fisher Scientific). Chromatographic separation was conducted on a Kinetex F5 column (2.1 ⅹ 150 mm, 2.6 µm particle size, 100 Å pore size, Phenomenex, Torrance, CA) with a binary gradient: solvent A was 0.1% formic acid in water and solvent B was 0.1% formic acid in acetonitrile. The gradient lasted for 10 minutes, starting at 2% solvent B, increasing to 25% B by 5.5 minutes, to 90% B by 6.5 minutes, holding at 90% B for 1 minute, then returning to 2% B in 0.3 minutes and maintaining for 2.2 minutes. The flow rate was 400 µL/min, with the autosampler and column temperature set at 4 ℃ and 35 ℃, respectively.

The electrospray ionization settings were as follows: sheath gas flow rate at 60 arbitrary units, auxiliary gas flow rate at 15 arbitrary units, sweep gas flow rate at 1 arbitrary units, spray voltages at 4.5 kV for positive mode, ion transfer tube temperature at 320 ℃, and vaporizer temperature at 340 ℃. For mass spectrometry, collision gas pressure was set at 1.5 mTorr, Q1 and Q3 resolutions were set to 0.7, and the cycle time was 0.5 seconds. Acetylcholine, dopamine, glutamate, and GABA were analyzed in positive mode with the following transitions: 146.1 m/z to 43, 58, and 87.1 m/z for acetylcholine; 154.2 m/z to 91, 119, and 137 m/z for dopamine; 148 m/z to 56, 84, and 102 m/z for glutamate; and 104.1 m/z to 43, 45, 69, and 87 m/z for GABA.

*9. Induction of nicotine withdrawal*

The mouse model of precipitated nicotine withdrawal was generated as in previous studies [5a, 5b], with minor modifications [5c]. (-)-Nicotine ditartrate was dissolved in physiological saline (0.175 mg/kg, free-base nicotine) and the pH was matched to 7.4. Mecamylamine hydrochloride was dissolved in physiological saline (3.0 mg/kg). Mice were intraperitoneally injected with the nicotine solution once/day for a duration of 3 days and were intraperitoneally injected with the mecamylamine solution at the 4^th^ day to precipitate the behavioral signs of nicotine withdrawal. This protocol for modeling nicotine withdrawal was sufficient to induce the physical and affective signs of nicotine withdrawal, as demonstrated in our behavioral data. Procyclidine hydrochloride was dissolved in physiological saline (3.0 or 10.0 mg/kg). Mice were intraperitoneally injected with the procyclidine solution, 30 min prior to the precipitation of nicotine withdrawal. For all drugs and vehicle, injection volume was 10 ml/kg.

*10. Chemogenetic inhibition*

To enforce chemogenetic inhibition of striatal ChIs, clozapine N-oxide (CNO) (#6329; Tocris Bioscience) was intraperitoneally injected into ChAT-Cre mice expressing hM4Di DREADD receptor in the striatal cholinergic interneurons. The CNO was dissolved in physiological saline and injected at 0.3 mg/kg at 30 min prior to the induction of nicotine withdrawal and measurement of somatic withdrawal signs.

*11. Behavior*

Mice were handled for more than 3 days (10 min/day) prior to behavioral tests. All behavioral tests were video-recorded for analysis. Nestlet shredding test and marble burying test were performed with the same batch of animals. For all other behaviors, tests were performed with an independent batch of animals.

*11.1. Somatic signs of nicotine withdrawal*

Somatic signs of nicotine withdrawal were measured as previously reported [5c]. A clear plexiglass column consisting of (in cm; L x W x H) 7 x 7 x 30 inner dimension with openings at the top and bottom was used for measurement of the somatic signs of nicotine withdrawal in mice. The floor luminosity was maintained at 100 lux. Immediately after the precipitation of nicotine withdrawal, mice were confined in the plexiglass column for 30 min to allow for a close-up video-examination of paw and body movements.

Following prior studies, the three most replicable and clinically translatable somatic signs of nicotine withdrawal were selected for measurements; paw tremor, body shakes, and freezing [5a-c, 6]. The number of events were counted for each sign: paw tremor (rapidly shaking paw(s) for two times while the two paws are supported on the ground or columnar wall, or three times while three paws are in support) (paw movement frequency >10 Hz, amplitude (peak-to-peak) >5 mm as defined previously [7]), body shakes (wet-dog shakes; rapidly shaking the body with the anteroposterior axis as the axis of rotation) (body shaking frequency ~30 Hz as defined previously [8]), and freezing (continuous immobility with minimal movement (head nodding or body trembling) and without paw movement for 60 seconds). For paw tremors or body shakes, 1) the events that occurred within 10 seconds of each other were counted as a single event (10-second epoch), and 2) the events that appeared 3 seconds before or after grooming were excluded from analysis (counted as an innate sequence for grooming).

*11.2. Nicotine conditioned place preference*

The nicotine conditioned place preference (CPP) test was conducted to measure the response to nicotine reward [9]. A three-chambered apparatus consisting of a white chamber, center zone, and black chamber was used for the nicotine CPP test. The luminosity of the white chamber, center zone, and black chamber were respectively maintained at 3~4, 300, and 10~12 lux to minimize side preference. The CPP test consisted of three sessions: Pre-test, conditioning, and post-test.

The pre-test consisted of a single 15-min session on the 1^st^ day. Mice were placed in the center zone and the guillotine doors to the side chambers (white and black) were simultaneously opened. Mice were allowed to freely explore the three-chambered apparatus for 15 min. The biased model for CPP test was used, in which nicotine was paired with the less-preferred chamber.

The conditioning consisted of six 20-min sessions beginning on the 2^nd^ day and finishing at the end of the 4^th^ day (2^nd^~4^th^ day), with two sessions/day. Within a day, the inter-session interval was 6 hours. In a conditioning session, mice were injected with either nicotine solution (0.175 mg/kg) or vehicle, and were respectively placed in the less-preferred or more-preferred chamber for 20 min. The order of conditioning was as follows: Nicotine-vehicle-vehicle-nicotine-nicotine-vehicle.

The post-test consisted of a single 15-min session on the 5^th^ day. Mice were placed in the center zone and the guillotine doors to the side chambers (white and black) were simultaneously opened. Mice were allowed to freely explore the three-chambered apparatus for 15 min.

To measure nicotine CPP, the % time spent in the nicotine-paired chamber in the post-test minus the % time spent in the nicotine-paired chamber in the pre-test was calculated.

*11.3. Nicotine-induced locomotor depression*

The nicotine-induced locomotor depression test was conducted to measure the extent of physical tolerance to nicotine [10]. A white open field box consisting of (in cm) 40 x 40 x 40 inner dimension was used to measure nicotine-induced locomotor depression. The floor luminosity was maintained at 5 lux. Immediately after intraperitoneal injection of vehicle (saline, 10 ml/kg) or nicotine solution (0.175 mg/kg), mice were placed facing one side of the wall within the open field box, and were allowed to explore the box for 30 min. The distance moved and the time spent in the center zone was measured using EthoVision XT 11.5 (Noldus, Wageningen, Netherlands). Vehicle was injected at day 0, and the nicotine solution was injected once/day at day 1~4. Test was conducted at days 0, 1, and 4.

*11.4. Open field test*

The open field test was conducted to measure general locomotor activity and anxiety-like behavior [5c]. A white open field box consisting of (in cm; L x W x H) 40 x 40 x 40 inner dimension was used for open field test. The floor luminosity was maintained at 5 lux. Immediately after the intraperitoneal injection of vehicle or mecamylamine solution, mice were placed facing one side of the wall within the open field box, and allowed to freely explore the box for 30 min. The distance moved in the open field and the time spent in the center zone (20 x 20 cm) was measured using EthoVision XT 11.5 (Noldus, Wageningen, Netherlands).

*11.5. Sucrose preference test*

The sucrose preference test was conducted to measure the response to a natural reward [11]. A clean plastic cage was used for the sucrose preference test. Mice were habituated to drink water from two bottles for 3 days. Then, mice were habituated to drink 1% sucrose solution from two bottles for 2 days to avoid neophobia. Next, mice were subjected to water deprivation for 16 hours. Lastly, mice were submitted to sucrose preference test without food.

During the test, mice were allowed to drink from two preweighed bottles, one containing drinking water and another containing a 1% sucrose solution, for 2 hours. Then the bottles were weighed and the positions of the two bottles were switched, and mice were again allowed to drink from the two pre-weighed bottles for 2 hours. The total fluid intake (in ml) and the % sucrose preference were calculated for the whole duration of sucrose preference test.

*11.6. Rotarod test*

The rotarod test was conducted to measure motor coordination and motor skill learning [12, 13]. A rotarod apparatus with a capacity of holding 6 mice (ENV-577M; Med Associates, VT, USA) was used for the rotarod test. Mice were first habituated on an accelerating rotarod (from 3.5 to 35 rpm for 5 min) once prior to testing. Then, mice were subjected to rotarod test for 3 days, 3 trials/day, with a 2-hour inter-trial interval within a day. A trial consisted of the rotarod accelerating from 3.5 to 35 rpm over 5 min. Mean latency to fall was measured for each day.

*11.7. Nestlet shredding test*

The nestlet shredding test was conducted to measure repetitive behavior [14]. A clean plastic cage and a piece of nestlet (Ancare, NY, USA) were used for nestlet shredding test. The floor luminosity was maintained at 50 lux. A single piece of nestlet was weighed, then placed into a clean plastic cage with minimal bedding and without food and water. Mice were introduced into the cage with the nestlet, and were left undisturbed for 1 hour. After testing, untorn pieces of nestlet (weighing > 0.1 g) were picked up with forceps. Loose materials (torn parts and bedding attached to the untorn pieces of nestlet) were carefully removed, and untorn nestlets were dried overnight. The dried nestlets were weighed.

*11.8. Marble burying test*

Marble burying test was conducted to measure repetitive behavior [14]. A clean plastic cage and 15 glass marbles (1 cm diameter) were used for the marble burying test. The floor luminosity was maintained at 200 lux. Marbles were evenly distributed in a layout of 3 x 5 on a clean cage filled with Beta Chip (Northeastern Products, NY, USA) 3 cm above ground level, and without food and water. Mice were introduced into the cage and were left undisturbed for 20 min. In 5-min intervals, the number of buried marbles was counted. A marble was scored as buried when > 2/3 of the marble’s surface area had been covered by Beta Chip.

*11.9. Y-maze spatial memory test*

Y-maze spatial memory test was conducted to measure spatial reference memory [15], with modifications. A black Y-maze with intramaze visual cues was used for the Y-maze spatial memory test. The floor luminosity was maintained at 200 lux. The Y-maze spatial memory test consisted of two sessions: Training and test.

The training consisted of 3-min sessions, two sessions/day, on day 1~3. Within a day, the inter-session interval was 6 hours. During the training, the entry to the “Novel” arm was blocked with a door. Within a training session, mice were placed in the “Start” arm and were allowed to freely explore the “Start” and “Other” arms for 3 min.

The test consisted of a single 10-min session on the 4^th^ day. The entry to “Novel” arm was permitted. In the test session, mice were placed in the “Start” arm and allowed to freely explore all three arms of the Y-maze for 10 min.

The number of entries to “Novel” and “Other” arms were counted. Discrimination index was calculated as the number of entries to “Novel” arm divided by the number of entries to “Novel” and “Other” arms.

*12. qPCR*

Camk2a-Cre mice were used to validate the Cre-dependent overexpression of miR-137 *in vivo*. AAV microinjection into the dorsal striatum of Camk2a-Cre mice yields a larger number of infected cells, compared to ChAT-Cre mice in which the cholinergic interneurons exist in sparse density in the dorsal striatum.

Total RNA from the AAV-injected dorsal striatum of Camk2a-Cre mice were extracted using TRIzol (Thermo Fisher Scientific) according to the manufacturer’s protocol. The yield and quality of RNA were determined using NanoDrop^TM^ 2000 (Thermo Fisher Scientific).

TaqMan™ MicroRNA Assay hsa-miR-137 (Thermo Fisher Scientific) was used for the quantification of miR-137 as previously reported [13]. cDNA was amplified from 50 ng of total RNA using TaqMan Universal Master Mix II, no UNG (Thermo Fisher Scientific) according to the manufacturer’s protocol. Then, mature miR-137 was quantified through qPCR with iQ SYBR Green Supermix (Bio-Rad, CA, USA). qPCR reactions were incubated on CFX Connect Real-Time PCR Detection System (Bio-Rad). All qPCR reactions were performed in triplicates. The relative abundance of miR-137 was calculated by the 2–ΔΔCt method. snoRNA202 was used as the normalization control.

*13. Western blot*

Fresh-frozen brain samples were coronally cryosectioned to a series of 150-μm sections, and the dorsal striatum was dissected out using surgical knife as in a previous study [13]. Total protein from the AAV-injected dorsal striatum of ChAT-Cre mice were extracted using the RIPA buffer (Thermo Fisher Scientific) according to the manufacturer's protocol. The yield of the protein was quantified using Protein Assay Dye Reagent Concentrate (Bio-Rad).

50 μg of protein sample was separated based on mass using SDS-PAGE. The separated protein sample was transferred onto a PVDF membrane. The membrane was subsequently blocked with 5% skim milk in 1X TBS-T (0.1% Tween-20), then washed and incubated in blocking buffer at 4 °C overnight with primary antibodies: Goat anti-ChAT (1:1000) (AB144P; Millipore, MA, USA), rabbit anti-choline transporter (1:200) (ACT-001; Alomone Labs, Jerusalem, Israel), or mouse anti-β-actin (1:500) (Santa Cruz Biotechnology, CA, USA). Next, the membrane was washed and incubated for 2 hours at room temperature with secondary antibodies: Donkey anti-goat IgG-HRP (1:5000) (Thermo Fisher Scientific), donkey anti-rabbit IgG-HRP (1:5000), or donkey anti-mouse IgG-HRP (1:5000). Finally, sections were washed and the HRP signal was developed using SuperSignal West Pico Chemiluminescent Substrate (Thermo Fisher Scientific) and visualized using Image Quant LAS4000 (GE Healthcare Bio-Sciences, Uppsala, Sweden).

Densitometric comparison was conducted between samples using ImageJ (National Institute of Health, Bethesda, MD, USA). Samples in the same blot were processed in parallel, and the loading control (β-actin) was run on the same blot as the targets. The loading control did not differ between groups.

*14. Cholinesterase assay*

Stock solution consisted of (in g/L) 4.1 sodium acetate anhydrous (S2889; Sigma-Aldrich), 1 cupric sulphate (209198; Sigma-Aldrich), and 1.2 glycine (G8898; Sigma-Aldrich) dissolved in distilled water and pH matched to 5.0. The stock solution was made at least 3 days prior to the cholinesterase assay experiment.

Mice were anesthetized with Avertin. Fresh-frozen brain samples were coronally cryosectioned to a 40-μm thickness in a -20 °C cryostat. Sections were mounted onto a slide glass and dried at room temperature for 15 min. The slide was incubated overnight at room temperature in an incubation solution consisting of (in mg/ml) 1.16 S-Acetylthiocholine iodide (#01480; Sigma-Aldrich) and 0.03 ethopropazine hydrochloride (E5406; Sigma-Aldrich) in stock solution. Then the slide was rinsed in distilled water for 5 min and incubated for 1 min in the developer solution consisting of 1% sodium sulfide nonahydrate (208043; Sigma-Aldrich), pH adjusted to 7.5 with 10% glacial acetic acid. Then the slide was rinsed in distilled water and was fixed in 3% PBS-buffered formalin overnight. Lastly, the slide was incubated in 100% ethanol for 5 min, then was incubated in xylene for 30 min, and mounted with permount/xylene solution (1:1 mixture).

The assay result was visualized with brightfield microscope and cholinesterase activity was quantified through ImageJ.

*15. In vivo microdialysis*

Mice were handled for more than 3 days (10 min/day) prior to microdialysis. At the day of experiment, mice were inserted through the cannula with microdialysis probe (MD-2211; BASi). The probe was connected to a 1.0 ml gastight syringe (MDN-0100; BASi) controlled via a programmable syringe drive (MD-1001; BASi). The no-net-flux (zero-net-flux) microdialysis method was implemented [16], in which the ACSF containing different concentrations of neurotransmitters that match the anticipated concentration of the same in the extracellular space of the dorsal striatum was perfused to the microdialysis probe and the amount of neurotransmitter analyte gained/lost from the microdialysis session was determined [17]. The ACSF for microdialysis was composed of (in mM) 124 NaCl, 2.5 KCl, 2 CaCl_2_, 2 MgCl_2_, 1.25 NaH_2_PO_4_, 20 HEPES, 0.25 ascorbic acid, and (in μM) 4 acetylcholine chloride, 2 dopamine hydrochloride, 50 GABA, and 200 L-glutamic acid with pH matched to 7.4. Microdialysis samples were collected every 20 min, at a rate of 1.0 μl/min.

Acetylcholine, dopamine, GABA, and glutamate were analyzed online by the HPLC Chromaster (Hitachi, Tokyo, Japan) coupled to DECADE Elite ECD (Antec Scientific, Zoeterwoude, Netherlands). For dopamine measurement, PCA buffer containing 2 mM EDTA and 20% perchloric acid (PCA) in distilled water was made prior to use. 1 μl of the PCA buffer was added to 19 μl of the microdialysis sample immediately after sample collection. For acetylcholine measurement, C18 HPLC column (#21455; Tosoh, Tokyo, Japan) was coupled to an AChE/ChOx immobilized enzyme reactor (IMER) (250.3532; Antec Scientific). For GABA and glutamate measurement, OPA buffer containing 16.4 mM o-Phthalaldehyde (OPA) (P0657; Sigma-Aldrich), 50 mM sodium sulfite, and 5% methanol in pH 10.4 sodium borate buffer was made prior to use. The OPA buffer, 1M NaOH, and the microdialysis sample were mixed at a ratio of 1:1:10, respectively, and incubated for more than 3 hours before analysis.

*16. Statistical analysis*

Experimental data were not pre-processed. All experiments were replicated at least once. Replication attempts were successful and observed measurements were consistent. All animals were randomly assigned into control or treatment group. Somatic withdrawal signs were analyzed blinded to the experimental conditions.

Two-tailed student’s *t*-test, and one-way analysis of variance (ANOVA) or two-way ANOVA followed by Holm-Sidak’s *post-hoc* test were conducted when appropriate. For ANOVA, ordinary or repeated measures (RM) was used as appropriated. *p* < 0.05 was considered statistically significant. Exact *p*-values and sample sizes (n) are indicated throughout the Results section and figure legends. Data were displayed as mean ± standard error of the mean (SEM). Statistical analyses were performed with Prism v6.0 (GraphPad, CA, USA).

Statistical significance in the comparisons of individual current steps in MEA data were summarized in Table S1.

**References**

[1] V. Agarwal, G. W. Bell, J. W. Nam, D. P. Bartel, *eLife* **2015**, *4*, e05005.

[2] M. D. Paraskevopoulou, G. Georgakilas, N. Kostoulas, I. S. Vlachos, T. Vergoulis, M. Reczko, C. Filippidis, T. Dalamagas, A. G. Hatzigeorgiou, *Nucleic Acids Research* **2013**, *41* (W1), W169, <https://doi.org/10.1093/nar/gkt393>.

[3] Acute Brain Slice Protocol, <https://www.axionbiosystems.com/resources/culture-protocol/acute-brain-slice-protocol>, accessed: **2024**.

[4] a) P. Varona, J. M. Ibarz, L. Lopez-Aguado, O. Herreras, *Journal of Neurophysiology* **2000**, *83* (4), 2192, <https://doi.org/10.1152/jn.2000.83.4.2192>; b) P. Andersen, T. Bliss, K. Skrede, *Experimental Brain Research* **1971**, *13* (2), 208.

[5] a) M. I. Damaj, W. Kao, B. R. Martin, *Journal of Pharmacology and Experimental Therapeutics* **2003**, *307* (2), 526, <https://doi.org/10.1124/jpet.103.054908>; b) R. Isola, V. Vogelsberg, T. A. Wemlinger, N. H. Neff, M. Hadjiconstantinou, *Brain Research* **1999**, *850* (1-2), 189, <https://doi.org/10.1016/s0006-8993(99)02131-9>; c) B. Kim, H.-I. Im, *Behavioral and Brain Functions* **2024**, *20*, 1, <https://doi.org/10.1186/s12993-024-00227-0>.

[6] D. H. Malin, J. Ronald Lake, V. A. Carter, J. Scott Cunningham, K. M. Hebert, D. L. Conrad, O. B. Wilson, *Psychopharmacology* **1994**, *115*, 180.

[7] S.-H. Kuo, E. D. Louis, P. L. Faust, A. Handforth, S.-y. Chang, B. Avlar, E. J. Lang, M.-K. Pan, L. N. Miterko, A. M. Brown, *The Cerebellum* **2019**, *18*, 1036.

[8] A. K. Dickerson, Z. G. Mills, D. L. Hu, *Journal of the Royal Society Interface* **2012**, *9* (77), 3208.

[9] a) S. Agatsuma, M. Lee, H. Zhu, K. Chen, J. C. Shih, I. Seif, N. Hiroi, *Human Molecular Genetics* **2006**, *15* (18), 2721, <https://doi.org/10.1093/hmg/ddl206>; b) R. E. Bernardi, R. Spanagel, *Drug and Alcohol Dependence* **2013**, *133* (2), 733; c) C. Peng, S. E. Engle, Y. Yan, M. M. Weera, J. N. Berry, M. C. Arvin, G. Zhao, J. M. McIntosh, J. A. Chester, R. M. Drenan, *PLoS One* **2017**, *12* (7), e0182142.

[10] C. Abburi, S. L. Wolfman, R. A. Metz, R. Kamber, D. S. McGehee, J. McDaid, *ENeuro* **2016**, *3* (4).

[11] M.-Y. Liu, C.-Y. Yin, L.-J. Zhu, X.-H. Zhu, C. Xu, C.-X. Luo, H. Chen, D.-Y. Zhu, Q.-G. Zhou, *Nature protocols* **2018**, *13* (7), 1686.

[12] B. Kim, H. I. Im, *Addiction Biology* **2021**, *26* (3), e12956.

[13] B. Kim, S. H. Tag, E. Nam, S. Ham, S. Ahn, J. Kim, D.-W. Cho, S. Lee, Y.-S. Yang, S. E. Lee, *Acta Pharmaceutica Sinica B* **2022**, *12* (8), 3281.

[14] M. Angoa-Pérez, M. J. Kane, D. I. Briggs, D. M. Francescutti, D. M. Kuhn, *JoVE (Journal of Visualized Experiments)* **2013**, (82), e50978.

[15] A.-K. Kraeuter, P. C. Guest, Z. Sarnyai, *Pre-clinical models: Techniques and protocols* **2019**, 105.

[16] V. I. Chefer, A. C. Thompson, A. Zapata, T. S. Shippenberg, *Current Protocols in Neuroscience* **2009**, *47* (1), 7.1. 1.

[17] J. Justice Jr, *Journal of neuroscience methods* **1993**, *48* (3), 263.
